# Anticancer Effect of Ginger Extract against Pancreatic Cancer Cells Mainly through Reactive Oxygen Species-Mediated Autotic Cell Death

**DOI:** 10.1371/journal.pone.0126605

**Published:** 2015-05-11

**Authors:** Miho Akimoto, Mari Iizuka, Rie Kanematsu, Masato Yoshida, Keizo Takenaga

**Affiliations:** 1 Laboratory of Tumor Biology, Department of Life Science, Shimane University Faculty of Medicine, Izumo, Shimane, Japan; 2 Laboratory of Molecular Science, Department of Life Science, Shimane University Faculty of Medicine, Izumo, Shimane, Japan; Complutense University, SPAIN

## Abstract

The extract of ginger (*Zingiber officinale Roscoe*) and its major pungent components, [6]-shogaol and [6]-gingerol, have been shown to have an anti-proliferative effect on several tumor cell lines. However, the anticancer activity of the ginger extract in pancreatic cancer is poorly understood. Here, we demonstrate that the ethanol-extracted materials of ginger suppressed cell cycle progression and consequently induced the death of human pancreatic cancer cell lines, including Panc-1 cells. The underlying mechanism entailed autosis, a recently characterized form of cell death, but not apoptosis or necroptosis. The extract markedly increased the LC3-II/LC3-I ratio, decreased SQSTM1/p62 protein, and enhanced vacuolization of the cytoplasm in Panc-1 cells. It activated AMPK, a positive regulator of autophagy, and inhibited mTOR, a negative autophagic regulator. The autophagy inhibitors 3-methyladenine and chloroquine partially prevented cell death. Morphologically, however, focal membrane rupture, nuclear shrinkage, focal swelling of the perinuclear space and electron dense mitochondria, which are unique morphological features of autosis, were observed. The extract enhanced reactive oxygen species (ROS) generation, and the antioxidant N-acetylcystein attenuated cell death. Our study revealed that daily intraperitoneal administration of the extract significantly prolonged survival (P = 0.0069) in a peritoneal dissemination model and suppressed tumor growth in an orthotopic model of pancreatic cancer (P < 0.01) without serious adverse effects. Although [6]-shogaol but not [6]-gingerol showed similar effects, chromatographic analyses suggested the presence of other constituent(s) as active substances. Together, these results show that ginger extract has potent anticancer activity against pancreatic cancer cells by inducing ROS-mediated autosis and warrants further investigation in order to develop an efficacious candidate drug.

## Introduction

Pancreatic cancer is a highly aggressive neoplasm with many chemotherapy- and radiotherapy-resistant phenotypes. The incidence of pancreatic cancer is increasing annually worldwide, becoming the fourth most common cause of cancer-related death in the world. Indeed, the estimated number of new pancreatic cancer cases was 277,000 in 2008 and 338,000 in 2012, and there were 266,000 pancreatic cancer deaths in 2008 and 331,000 pancreatic cancer deaths in 2012 [1, 2]. As the majority of pancreatic cancer patients are diagnosed at an inoperable stage [3, 4], the overall 5-year relative survival rate is low, and the median survival time is only 6 months even in patients receiving quality treatment. In order to obtain new leads for the development of preventive and therapeutic strategies, many research efforts have been focused on understanding the molecular mechanisms underlying pancreatic cancer progression [3, 4].

Ginger (*Zingiber officinale Roscoe*), a rhizomatous perennial plant, is widely used as a spice in foods and beverages and utilized primarily as a remedy for digestive disorders including dyspepsia, nausea, gastritis, vomiting, colic, and diarrhea [5, 6]. Ginger extract and its pungent components, such as [6]-gingerol and [6]-shogaol, are also known to exhibit many biological effects including anti-inflammation, antioxidation and anticancer activity [5, 6]. Because of its strong anti-inflammatory activity, ginger has recently drawn attention as a remedy for osteoarthritis and rheumatoid arthritis [7, 8]. With respect to anticancer activity, ginger and its constituents have been shown to inhibit the proliferation of and induce apoptosis of a variety of cancer cell types *in vitro* [9–15]. In addition, the use of ginger for the chemoprevention of colorectal cancer has attracted attention [16–18]. However, the anticancer activity of ginger extract and its constituents against pancreatic cancer has been poorly investigated.

In this study, we examined the anticancer activity of ginger extract against pancreatic cancer cells both *in vitro* and *in vivo* and investigated its potential mechanism. Here, we report that ginger extract leads to the reduction of cell viability and tumor growth of Panc-1 cells mainly through ROS-mediated autosis, a recently characterized form of cell death.

## Materials and Methods

### Cells and cell culture

Human pancreatic cancer cells, Panc-1, AsPC-1, BxPC-3, CAPAN-2, CFPAC-1, MIAPaCa-2 and SW1990, and mouse pancreatic cancer cells, Panc02, were used in this study. Panc-1 and MIAPaCa-2 cells were obtained from the RIKEN BRC Cell Bank (Tsukuba, Japan), and other human pancreatic cell lines were purchased from ATCC (Manassas, VA). Panc02 cells were kindly provided by Dr. T. Hollingsworth, University of Nebraska Medical Center [19, 20]. Panc-1-Luc-ZsGreen cells and Panc02-Luc-ZsGreen cells expressing firefly luciferase and ZsGreen were established by lentiviral transduction of the plasmid pHIV-Luc-ZsGreen, which has been deposited with Addgene (http://www.addgene.org/Bryan_Welm/), and subsequent cloning. Human pulmonary alveolar epithelial cells (HPAEpiC) were purchased from ScienCell (Carlsbad, CA, USA) and were maintained in alveolar epithelial cell medium (AEpiCM) supplemented with 2% fetal bovine serum (FBS), epithelial cell growth supplement (EpiCGS) and penicillin/streptomycin. Human umbilical vein endothelial cells (HUVEC) were obtained from Lonza Walersville, Inc. (Walkersville, MD, USA) and were grown in EBM-2 supplemented with EGM SingleQuats (Lonza). Mitochondria DNA-less P29 (ρ^0^P29) cells and the cybrid P29mtP29 cells that were reintroduced with P29 mtDNA into ρ^0^P29 cells were established from Lewis lung carcinoma P29 cells [21]. Colon cancer cells (Colo320DM, HT29, LoVo, LS174T, SW480, SW620) [22], gastric cancer cells (MKN1, MKN45) [23], lung cancer cells (A549, QG56, PC-10, PC-1) [24], breast cancer cells (MCF7, BT549, MDA-MB-231, MDA-MB-468) [25, 26], leukemia cells (THP-1, K562) [27], osteosarcoma cells (Saos-2) [28], cervical cancer cells (HeLa) [29], hepatoma cells (HepG2) [30], fibrosarcoma cells (HT1080) [29], and mouse colon carcinoma LuM1 cells derived from colon 26 tumor [31] were also used in this study. HT29, LS174T, SW480, SW620 and MDA-MB-468 were purchased from ATCC. MCF7 and HT1080 cells were obtained from the JCRB Cell Bank. THP-1 and K562 cells were kindly provided by Dr. Y. Honma, Shimane University Faculty of Medicine. Gastric cancer cell lines were supplied by the Department of Pathology (Dr. S. Morikawa), Shimane University Faculty of Medicine. Other cell lines were supplied by Dr. A. Nakagawara, Chiba Cancer Center Research Institute. Characteristics of the cell lines used in this study are described elsewhere [19–31]. Leukemia cell lines were cultured in RPMI1640 medium containing 10% heat-inactivated fetal bovine serum (FBS) and 40 μg/ml gentamicin. Other cell lines were cultured in Dulbecco’s modified Eagle’s medium (DMEM) containing 10% FBS and 40 μg/ml gentamicin in a humidified atmosphere with 21% O_2_/5% CO_2_ (normoxia) or 1% O_2_/5% CO_2_ (hypoxia). Hypoxic culture conditions were achieved in a humidified automatic O_2_/CO_2_ incubator (Wakenyaku, Kyoto, Japan).

### Reagents

[6]-Shogaol and [6]-gingerol were purchased from TOKIWA PHYTOCHEMICAL CO., Ltd. (Chiba, Japan).

### Preparation of ginger extract (SSHE)

Dry powder of the root parts of Syussai Shoga (ginger in Japanese), which was cultivated in the Hikawa area in Izumo, Shimane prefecture, was extracted with ethanol (10:1; volume for weight) for 20 min in a sonication water bath. Ethanol was evaporated at 80°C to yield a crude ethanol extract of the ginger (referred to as SSHE). The extract was weighed and dissolved in ethanol or dimethylsulfoxide (DMSO) at the desired concentration.

### Cell growth and viability assay

Cell growth and viability was measured by using the MTT (3-(4,5-dimethylthiazol-2-yl)-2,5-diphenyltetrazolium bromide) assay. Briefly, cells (2x10^4^ cells/well) were cultured in 96-well tissue culture plates and treated in triplicate in 100 μl DMEM/10% FBS containing different concentrations of SSHE or solvent alone for the indicated period. At the end of the incubation, 10 μl of MTT (2.5 mg/ml) (Sigma-Aldrich Japan, Tokyo, Japan) was added to the wells to allow formation of MTT formazan crystals for 4 h. After the medium was removed, the crystals were solubilized in 100 μl of DMSO. Absorbance was recorded at 550 nm. Cell viability was also assayed by a trypan blue dye exclusion test.

### Cell cycle analysis

Panc-1 cells treated with SSHE for 20 h were fixed in 70% ethanol and stored at -20°C until use. The fixed cells were washed with Dulbecco’s PBS (DPBS) and incubated with 100 μg/ml RNase A and 50 μg/ml PI (Sigma-Aldrich Japan). The cells were then subjected to flow cytometric analysis using a FACSCalibur flow cytometer (BD Biosciences, Franklin Lakes, NJ).

### Measurement of mitochondria membrane potential

Mitochondria membrane potential was monitored by the JC-1 Mitochondrial Membrane Potential Assay Kit (Cayman Chemical, Ann Arbor, MI). For this assay, 100 μl/ml of the JC-1 Staining Solution was added to Panc-1 cells treated with SSHE for 20 h in 6-well plates, and the cells were incubated for 15 min. Afterwards, the cells were detached by trypsinization, washed twice with the assay buffer, and then subjected to flow cytometry.

### Annexin V/Propidium iodide (PI) staining

The Annexin V-FITC Apoptosis Detection Kit (BECKMAN COULTER Inc., Pasadena, CA) was used to detect annexin V and/or PI-positive cells. Briefly, Panc-1 cells were washed with ice-cold DPBS and then stained with Annexin V-FITC for 15 min at room temperature in the dark and PI in ice-cold Binding Buffer. Annexin V and/or PI-positive cells were counted using a FACS Calibur flow cytometer.

### Measurement of ROS generation

The production of ROS was monitored by flow cytometry with 2',7'-dichlorodihydrofluorescein diacetate (H2DCFDA) (Molecular Probe-Life Technologies, Carlsbad, CA) and mitochondrial superoxide indicator MitoSOX Red (Invitrogen) as probes. Briefly, Panc-1 cells treated with SSHE were incubated with 10 μM of H2DCFDA or 5 μM of MitoSOX Red in serum-free DMEM for 10 min. The medium was removed, and the cells were detached with a brief treatment of 0.25% trypsin in Hank’s balanced salt solution. After addition of fresh culture medium, the cells were collected by centrifugation, washed once with DPBS and suspended in DPBS. The fluorescence was monitored using the FACSCalibur flow cytometer or under a laser confocal microscope (Fluoview FV1000, Olympus, Tokyo).

### Western blotting

Cell extracts were prepared by lysing cells with RIPA buffer (50 mM Tris—HCl, pH 7.4, 150 mM NaCl, 1% NP-40, 0.5% deoxycholate, 0.1% sodium dodecyl sulfate, 2 mM EDTA, protease inhibitor cocktail and phosphatase inhibitor cocktail) on ice for 20 min. The lysates were centrifuged at 12,000 ×*g* for 10 min at 4°C,and the supernatants were used for subsequent analyses. SDS—polyacrylamide gel electrophoresis and immunoblot analyses were performed as described previously [32]. The primary antibodies used were rabbit monoclonal anti-SQSTM1/p62 (D5E2, 1:1,000 dilution, Cell Signaling Technology, Danvers, MA), rabbit polyclonal anti-LC3B (1:1,000 dilution, Cell Signaling), rabbit polyclonal anti-phospho-mTOR (S2481) (1:1,000 dilution, Cell Signaling), rabbit monoclonal anti-mTOR (7C10, 1:1,000 dilution, Cell Signaling), rabbit monoclonal anti-phospho-AMPKα (T172) (40H9, 1:1,000 dilution, Cell Signaling), and rabbit monoclonal anti-AMPKα antibody (23A3, 1:1,000 dilution, Cell Signaling). The secondary antibodies were HRP-conjugated rabbit or anti-mouse IgG (1:3,000 dilution, Cell Signaling). For loading controls, anti-β-actin antibody (sc-47778, 1:3,000 dilution, Santa Cruz Biotechnology) was used. Signals were visualized using ECL plus (GE Healthcare, Little Chalfont, UK). The membranes were scanned with a Luminoimaging Analyzer LAS4000 (GE Healthcare).

### Immunofluorescent staining

Panc-1 cells treated with solvent alone or SSHE were fixed with 4% formaldehyde/5% sucrose in DPBS for 20 min, rinsed with DPBS, and permeabilized with 0.5% Triton X-100 in DPBS for 4 min. In some experiments, cells were stained with 100 nM of MitoTracker Red CMXRos (Invitrogen) for 10 min before fixation. The cells were blocked with 3% BSA/0.1% glycine in DPBS for 1 h, rinsed, and then incubated with rabbit monoclonal anti-AIF (D39D2, 1:200 dilution, Cell Signaling) or rabbit polyclonal anti-LC3B antibody (1:200 dilution) for 1 h. After extensive washing with DPBS, the cells were incubated with Alexa Fluor 488-conjugated goat anti-rabbit IgG (1:300 dilution, Invitrogen) for 1 h. The cells were counterstained with DAPI and observed under a laser scanning confocal microscope.

### Transmission electron microscopy (TEM) analysis

Untreated Panc-1 cells and the cells treated with 200 μg/ml SSHE for 28 h were placed in 2% paraformaldehyde and 2% glutaraldehyde in 30 mM HEPES buffer (30 mM HEPES, 100 mM NaCl, 2 mM CaCl_2_, pH 7.4) for 2 h at 4°C and post-fixed with 1% OsO_4_ for 1 h at 4°C. The samples were dehydrated with graded alcohol and embedded in TAAB812 resin (TAAB Laboratories Equipment Ltd., Berkshire, UK). Ultrathin sections were stained for 1 h in 3% aqueous uranyl acetate, washed, and counterstained with 0.3% lead citrate, and they were examined on a transmission electron microscope (EM-002B, JEOL Ltd., Tokyo, Japan).

### GFP-LC3 plasmid and transfection

EGFP-LC3B fusion plasmid (pCMX-SAH/Y145F-LC3B-GFP) was constructed by cloning LC3B cDNA, which was amplified by PCR, into a pCMX-SAH/Y145F-GFP vector [33]. The construct was verified by DNA sequencing. Panc-1 ells were transiently transfected with the plasmid using Lipofectamine 2000 (Invitrogen-Life Technologies, Carlsbad, CA) according to the manufacturer’s instructions. After 24 h, the cells were exposed to SSHE and examined under a laser scanning confocal microscope.

### Animal experiments

All animal experiments were performed in compliance with the institutional guidelines for the care and use of animal research. The protocol was approved by the IZUMO Campus Animal Care and Use Committee of Shimane University (Permission Number: IZ26-7). All the mice were housed in the animal center of Shimane University Faculty of Medicine under specific pathogen-free conditions at a controlled temperature of 23±2°C, relative humidity 55±10%, and with 12 h light/12 h dark cycles. They were given food and water *ad libitum*. Mice were checked for their health during the entire experimental period at least once a day after tumor injection. All surgery was performed under medetomidine (0.3 mg/kg)/midazolam (4.0 mg/kg)/butorphanol (5.0 mg/kg) anesthesia. Mice were normally euthanized by CO_2_ inhalation at the end of a study. For the peritoneal dissemination model, Panc02-Luc-ZsGreen cells (5x10^5^ cells/mouse) were injected intraperitoneally as a cell suspension into 7-week-old male C57BL/6 mice (Crea Japan, Tokyo, Japan), and the mice were randomized and grouped into the control (n = 8) and the SSHE groups (n = 8). The treatment regimens were started the day after tumor inoculation. Mice were euthanised in a CO_2_ chamber when they were moribund, measured by a lack of sustained purposeful response to gentle stimuli. All efforts including subcutaneous administration of meloxicam (5 mg/kg) were made to minimize suffering. The experiment was performed twice, and the combined data were subjected to analyses. For the orthotopic model of pancreatic cancer, Panc-1-Luc-ZsGreen cells (1x10^6^ cells/mouse) were implanted with 50% Matrigel into the pancreas of 6-week-old female nude mice (BALB/c nu/nu, Japan SLC, Shizuoka, Japan) [32]. One week after the injection, they were randomized and grouped into the control (n = 6) and the SSHE groups (n = 6). The treatment regimens were started one week after tumor injection. For mouse colon carcinoma experiments, LuM1 cells (3 x 10^5^ cells) were subcutaneously implanted in male Balb/c mice (n = 6 for the control and the SSHE group). The volumes of LuM1 tumors were evaluated by measuring two perpendicular diameters with calipers. Tumor volume (V) was calculated using the following equation: V = *(a* ^*2*^ *x b)/2*, where *a* is the small diameter and *b* the large diameter. In the SSHE group, mice were intraperitoneally administered 80 mg/kg SSHE once daily. In the control group, mice were administered solvent alone in DPBS.

### Bioluminescent imaging

*In vivo* bioluminescent imaging was performed using the IVIS imaging system (Caliper Life Sciences, Hopkinton, MA). All mice were injected intraperitoneally with 150 mg/kg d-luciferin (Promega, Fitchburg, WI) and anesthetized with 2.5% isoflurane. Ten minutes later, photons from animals’ whole bodies were imaged using the IVIS imaging system (Caliper Life Sciences) according to the manufacturer's instructions. Data were analyzed by living image 2.50 software (Caliper Life Sciences).

### Blood hematology and biochemistry test

Mice were anesthetized, and blood was collected from the heart. Peripheral blood profiles were analyzed by the Sysmex KX-21NV automated hematology analyzer (Sysmex, Kobe, Japan). The levels of white blood cells (WBC), red blood cells (RBC), platelets (PLT), hemoglobin (HGB), hematocrit (HCT), mean corpuscular volume (MCV), mean corpuscular hemoglobin (MCH), and mean corpuscular hemoglobin concentration (MCHC) were examined. Glucose (Glu) levels, total cholesterol (T-Cho) levels, alanine aminotransferase (ALT) and aspartate aminotransferase (AST) levels, and blood urea nitrogen (BUN) levels were analyzed with an automated analyzer for clinical chemistry, SPOTCHEM EZ SP-4430 (ARKRAY, Inc., Kyoto), using SPOTCHEM II test strips.

### Reversed-phase high-performance liquid chromatography (HPLC)

Liquid chromatographic separations were achieved using a reversed-phase C-18, 3 μm, 2.4 × 250 mm column (COSMOSIL. NAKARAI TESQUE, Inc., Kyoto, Japan) at a flow rate of 1 ml/min. The mobile phase was 70% methanol. The elution profile was monitored by UV spectrophotometry at 228 nm.

### Statistical analysis

All data are presented as the mean ± SD. Statistical significance between data sets was tested by unpaired Student’s *t* test. Survival of mice was analyzed using the log-rank test. P < 0.05 was considered to be statistically significant.

## Results

### SSHE inhibits cell cycle progression and induces the death of pancreatic cancer cell lines

**Fig 1:**
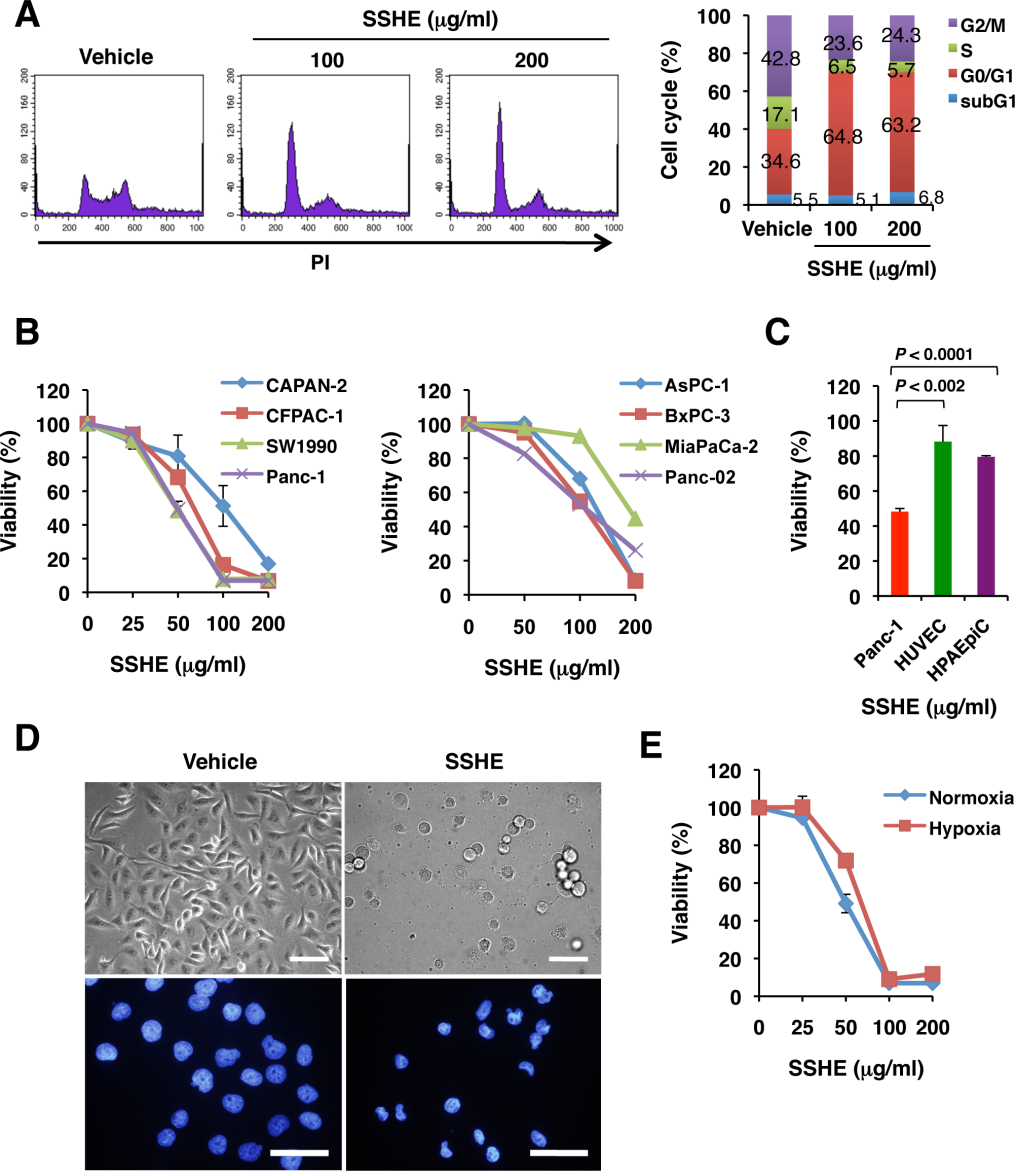
Effect of SSHE on cell cycle and viability of pancreatic cancer cell lines. (A) Cell cycle. Panc-1 cells were treated with vehicle alone, 100 μg/ml SSHE or 200 μg/ml SSHE for 20 h, fixed, stained with PI, and then subjected to cytometry. (B) Cell viability. Pancreatic cancer cell lines were treated with vehicle alone or various concentrations of SSHE for 42 h. Cell viability was assessed by the MTT assay. Bars; SD. (C) Effect of SSHE on normal cells. HUVEC, HPAEpiC and Panc-1 cells were treated with vehicle alone or 100 μg/ml SSHE for 38 h. Cell viability was assessed by the MTT assay. Bars; SD. (D) Morphology. Panc-1 cells were treated with vehicle alone (left) or 200 μg/ml SSHE for 40 h (right). (E) Effect of SSHE on Panc-1 cells under hypoxic conditions. Panc-1 cells were treated with vehicle alone or various concentrations of SSHE for 42 h. Cell viability was determined by the MTT assay.

Treatment of Panc-1 cells with SSHE for 20 h resulted in an arrest in the G0/G1 phase of the cell cycle (Fig 1A). An increase in the subG1 fraction, a feature of apoptosis, was marginal. SSHE eventually induced the death of Panc-1 cells and other human and mouse pancreatic cancer cell lines (Fig 1B). Normal cells such as HUVEC and HPAEpiC were more resistant to SSHE compared to Panc-1 cells (Fig 1C). At the later stages of cell death of Panc-1 cells, focal plasma membrane rupture and the shrinkage of the nucleus were apparent (Fig 1D). It should be noted that fragmentation of the nucleus, another feature of apoptosis, was rarely observed at this stage. SSHE was also effective in inducing the death of Panc-1 cells under hypoxic conditions (Fig 1E). It also caused a significant growth retardation and death in a variety of additional tumor cell lines, such as colon cancer, gastric cancer, lung cancer, breast cancer, leukemia, osteosarcoma, hepatoma, cervical cancer and fibrosarcoma (S1 Fig).

### SSHE induces autotic cell death rather than apoptosis or necroptosis in Panc-1 cells

**Fig 2:**
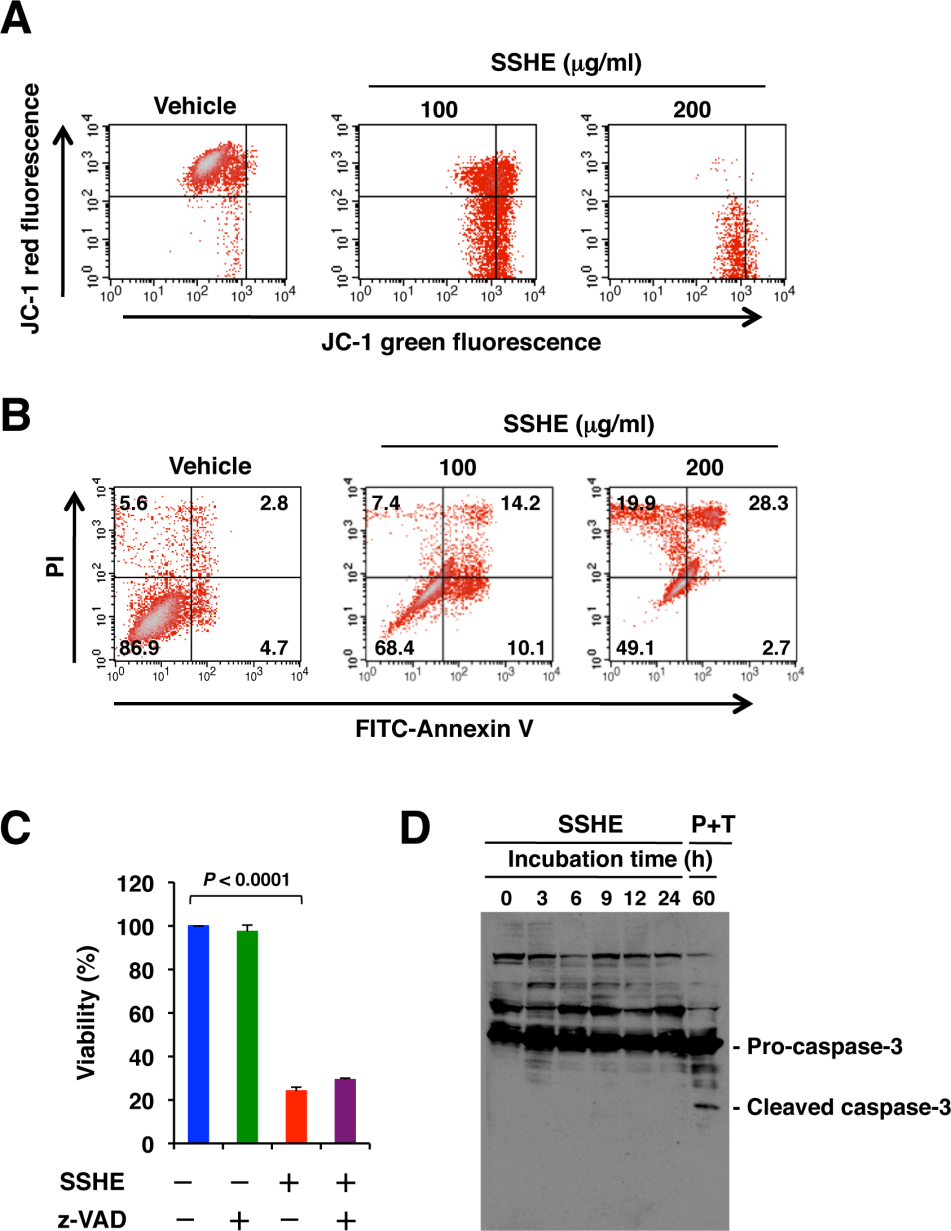
Effect of SSHE on the features of apoptosis in Panc-1 cells. (A) Mitochondrial membrane potential. Panc-1 cells were treated with vehicle alone, 100 μg/ml SSHE or 200 μg/ml SSHE for 20 h and then subjected to the JC-1 assay. Healthy cells with functional mitochondria that contain red JC-1 J-aggregates and apoptotic or unhealthy cells with collapsed mitochondria that contain mainly green JC-1 monomers were detected by cytometry. (B) Annexin V/PI staining. Panc-1 cells were treated with vehicle alone, 100 μg/ml SSHE or 200 μg/ml SSHE for 24 h and then subjected to annexin V/PI staining. (C) Effect of zVAD-fmk (zVAD) on SSHE-induced cell death. Panc-1 cells were incubated for 42 h. Cell viability was assessed by the MTT assay. Bars; SD. (D) Caspase-3 activation. Panc-1 cells were incubated with 200 μg/ml SSHE for various time periods or treated with 10 μM pifithrin-μ and 100 ng/ml TRAIL for 60 h (served as positive control). The cell lysates were subjected to Western blot analysis with anti-caspase 3 antibody.

To investigate the mechanism whereby SSHE induced the death of Panc-1 cells, we investigated various markers of apoptotic cells. The JC-1 assay revealed a marked reduction of mitochondria membrane potential after SSHE treatment (Fig 2A). Annexin V/PI staining also showed an increase in the percentage of annexin V-positive and/or PI-positive cells, depending on the concentration of SSHE (Fig 2B). However, the pan-caspase inhibitor zVAD-fmk did not ameliorate cell survival (Fig 2C). Furthermore, cleaved caspase 3 was hardly detectable after treatment with 200 μg/ml SSHE for 24 h. After SSHE treatment, nearly 30% of the cells were PI positive, whereas treatment of the cells with pifithrin-μ and TRAIL activated caspase-3 (Fig 2D), coinciding with a previous report [34]. Based on the data that suggest that SSHE neither increased the percentage of the subG1 fraction (Fig 1A) nor induced fragmentation of the nuclei (Fig 1D), we could not obtain concrete evidence of apoptosis in the SSHE-treated Panc-1 cells. Additionally, translocation of apoptosis-inducing factor (AIF), a mitochondrial caspase-independent death effector, into the nucleus was not apparent (S2 Fig), and necrostatin-1, an inhibitor of RIP1 kinase-mediated necroptosis, failed to prevent SSHE-induced cell death (S2 Fig). Thus, neither mitochondria-independent apoptosis nor necroptosis was involved in SSHE-induced cell death.

**Fig 3:**
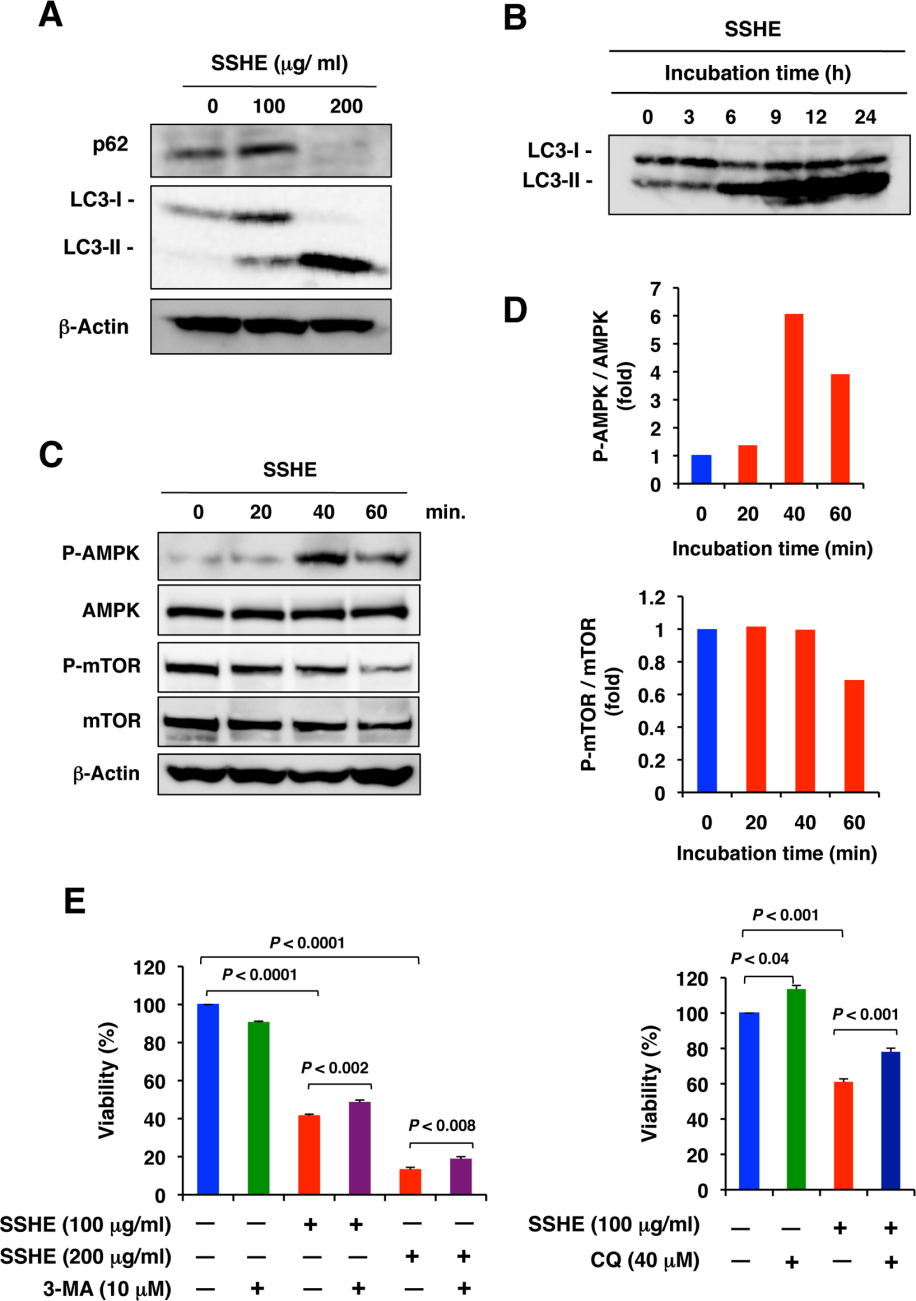
Effect of SSHE on the features of autophagy in Panc-1 cells. (A) Effect of SSHE on the expression of autophagy-related proteins. Panc-1 cells were treated with vehicle alone, 100 μg/ml SSHE or 200 μg/ml SSHE for 24 h. The cell lysates were subjected to Western blot analysis with anti-SQSTM1/p62 or anti-LC3 antibody. β-Actin served as a loading control. (B) Time-course of the conversion of LC3-I to LC3-II. Panc-1 cells were treated with 200 μg/ml SSHE for the indicated times. (C) AMPK and mTOR expression. Panc-1 cells were treated with 200 μg/ml SSHE for various times. The cell lysates were subjected to Western blot analysis with anti-AMPK, anti-phospho-AMPK antibody, anti-mTOR, or anti-phospho-mTOR. β-Actin served as a loading control. (D) AMPK activation and mTOR inhibition by SSHE. The intensity of the bands in (C) was quantified by Image J software. (E) Effect of 3-methyladenine (3-MA) and chloroquine (CQ) on SSHE-induced cell death. Panc-1 cells were treated with SSHE at the indicated concentration in the absence or presence of 10 μM 3-MA (left) or 40 μM CQ for 42 h. Cell viability was assessed by the MTT assay. Western blot images (A-C) have been cropped for presentation. Full size images are presented in S13 Fig Bars; SD.

**Fig 4:**
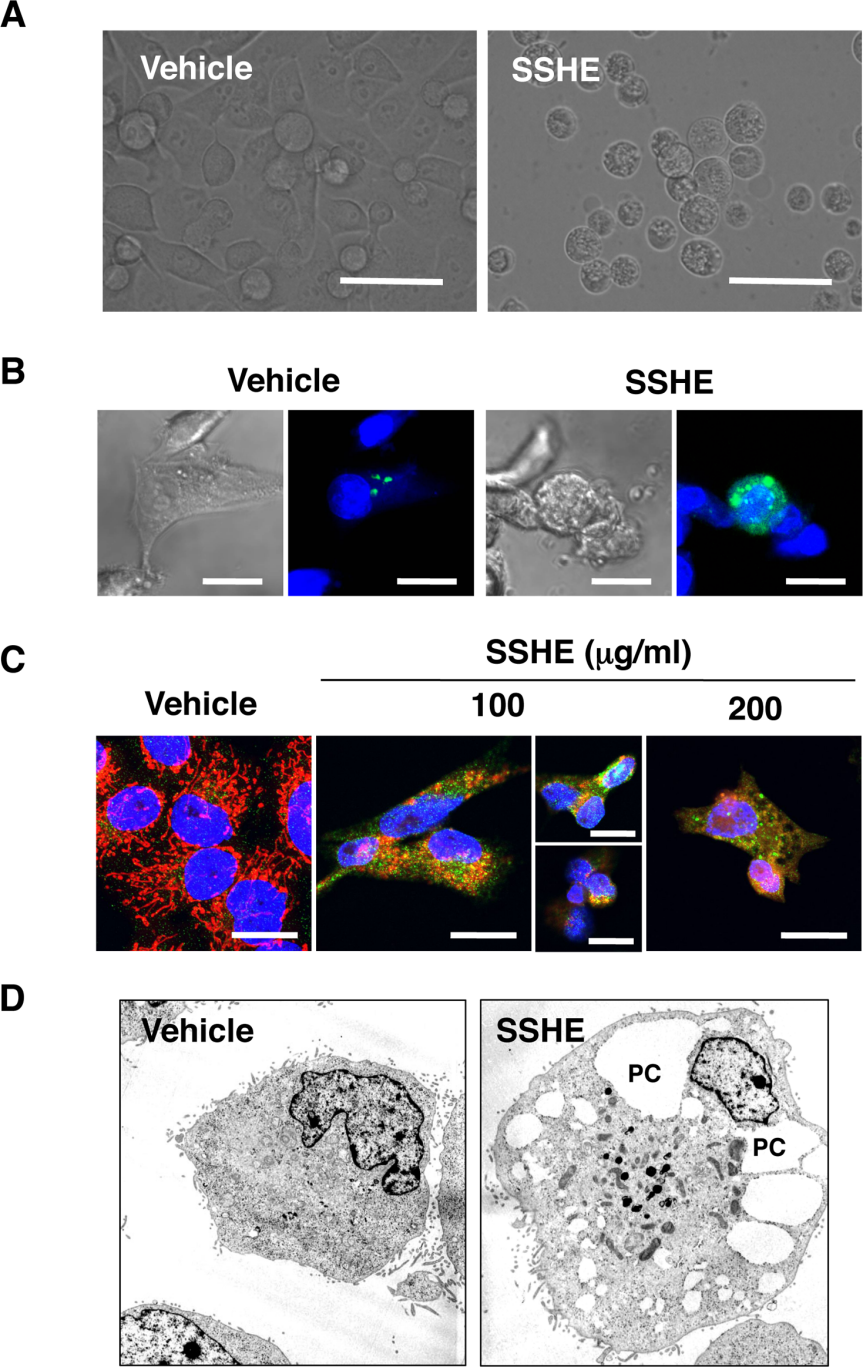
Effect of SSHE on the morphological features of Panc-1 cells. (A) Phase contrast. Panc-1 cells were treated with vehicle alone or 100 μg/ml SSHE for 40 h. Bars; 100 μm. (B) LC-3 puncta. Panc-1 cells transfected with pCMX-SAH/Y145F-LC3B-GFP vector were treated with vehicle alone or 100 μg/ml SSHE for 24 h and were observed under a confocal laser microscope. Bars; 20 μm. (C) Double staining with MitoTracker and anti-LC3 antibody. Panc-1 cells were treated with vehicle alone, 100 μg/ml or 200 μg/ml SSHE for 24 h, stained with 100 nM of MitoTracker Red for 10 min, fixed, and then processed for LC3 immunostaining. Bars; 20 μm. (D) Ultrastructure. Panc-1 cells treated vehicle alone or with 200 μg/ml SSHE for 28 h were processed for TEM analysis. Original magnification, x2,500. PC, perinuclear space.

On the other hand, SSHE markedly increased the LC3-II/LC3-I ratio, an indicator of autophagosome formation, in a dose- and time-dependent manner. SSHE also decreased SQSTM1/p62 protein levels, one of the specific substrates degraded through the autophagy-lysosomal pathway, in Panc-1 cells (Fig 3A and 3B). SSHE activated AMPK, a positive regulator of autophagy, and inhibited mTOR, a negative autophagic regulator (Fig 3C and 3D). The autophagy inhibitors 3-methyladenine and chloroquine partially prevented cell death (Fig 3E). Morphologically, SSHE-treated cells showed massive vacuolization of the cytoplasm approximately 24 h after treatment (Fig 4A). These cytoplasmic vacuoles were likely autophagosomes because GFP-LC3 puncta appeared after treatment with SSHE (Fig 4B). Some LC3 puncta were co-localized with MitoTracker-positive mitochondria in the cells treated with 100 μg/ml SSHE for 20 h, suggesting the occurrence of mitophagy (Fig 4C). Thus, SSHE-induced cell death seemed to be autophagic cell death. However, TEM analyses 28 h after SSHE treatment showed electron-dense mitochondria, empty vacuoles and focal perinuclear swelling (Fig 4D), which are not observed in classical type autophagy [35]. Thus, we concluded that SSHE-induced cell death was caspase-independent and resembled autophagy cell death. This form of cell death coincides well with autosis, a recently characterized Na^+^,K^+^-ATPase-regulated form of cell death [36]. Unfortunately, because the cardiac glycoside digoxin, an antagonist of Na^+^,K^+^-ATPase that is reported to inhibit autosis [36], was too cytotoxic to Panc-1 cells, we could not evaluate its effect on SSHE-induced cell death.

### Mitochondrial ROS generation is involved in SSHE-induced cell death

**Fig 5:**
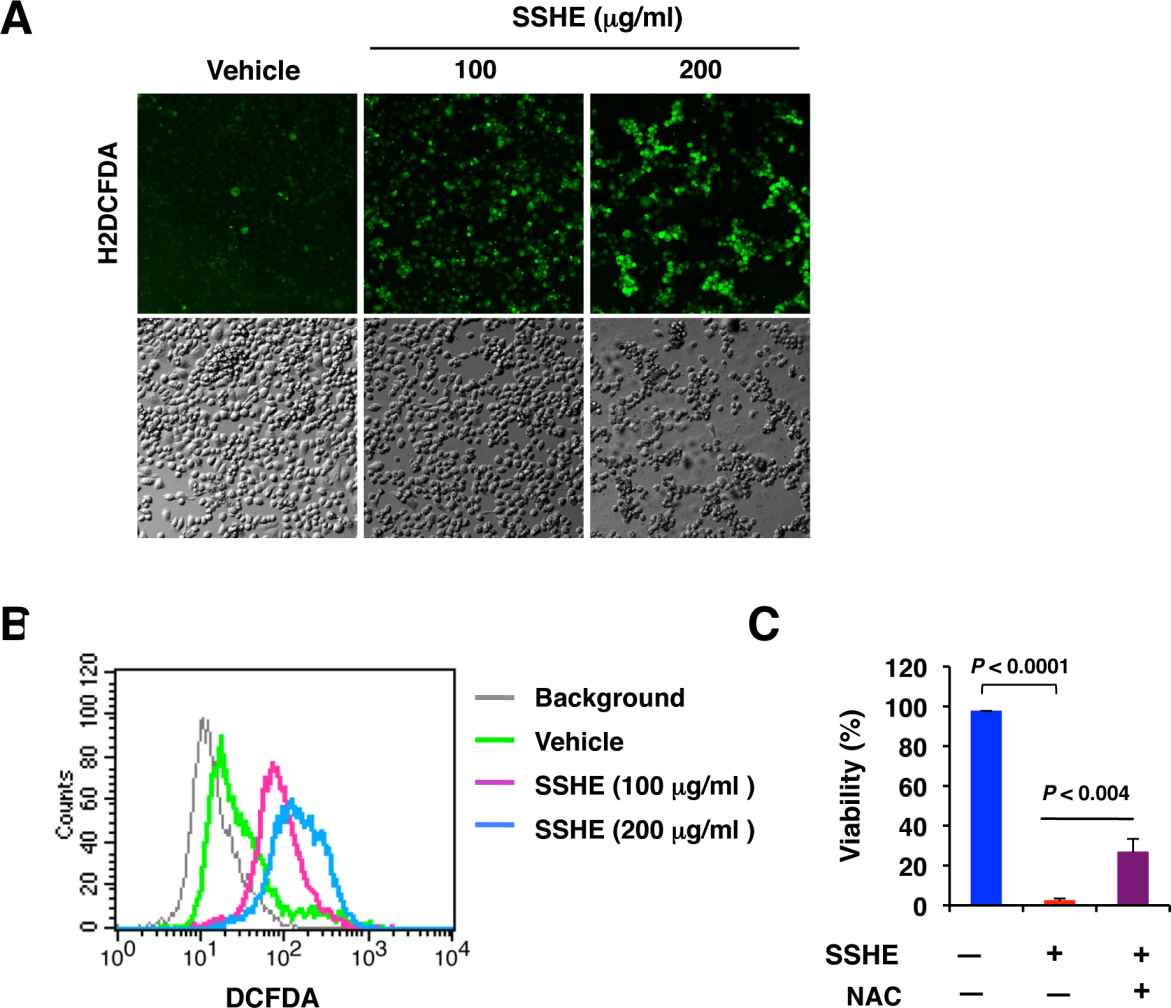
ROS production in SSHE-treated Panc-1 cells. (A) Panc-1 cells treated with vehicle alone, 100 μg/ml SSHE or 200 μg/ml SSHE for 20 h were stained with 10 μM H2DCFDA for 10 min and immediately observed under a confocal laser microscope. (B) Panc-1 cells treated as in A were subjected to cytometry. (C) Effect of NAC on SSHE-induced cell death. Panc-1 cells were treated with 200 μg/ml SSHE in the presence or absence of 10 mM NAC for 42 h. Cell viability was assessed by a trypan blue dye exclusion test. Bars; SD.

Changes in ROS generation, as assessed by H2DCFDA staining, after treatment of Panc-1 cells with SSHE showed a biphasic pattern. At the early stages (approximately 10 h), ROS generation was inhibited by SSHE (S3 Fig). However, prolonged treatment resulted in a robust increase in ROS generation (Fig 5A and 5B). MitoSOX Red staining also showed the increased production of mitochondrial superoxide (S4 Fig). The antioxidant N-acetylcystein (NAC) significantly attenuated the cell death induced by SSHE as evaluated by the trypan blue dye exclusion test (Fig 5C). These results suggested ROS generation as a cause of SSHE-induced cell death.

Mitochondria are reported to be the primary source of ROS required for autophagy induction [37]. To examine whether mitochondrial ROS play a role in SSHE-induced autotic cell death, we utilized mitochondrial DNA (mtDNA)-less ρ^0^P29 cells that are derived from Lewis lung carcinoma P29 cells and P29mtP29 cells that were established by reintroducing wild-type mtDNA into ρ^0^P29 cells. When ρ^0^P29 cells were treated with SSHE they scarcely produced ROS, while P29mtP29 cells sufficiently produced ROS (S5 Fig). Interestingly, ρ^0^P29 cells were revealed to be resistant to SSHE compared with P29mtP29 cells (S5 Fig).

### SSHE retards tumor growth of pancreatic cancer

**Fig 6:**
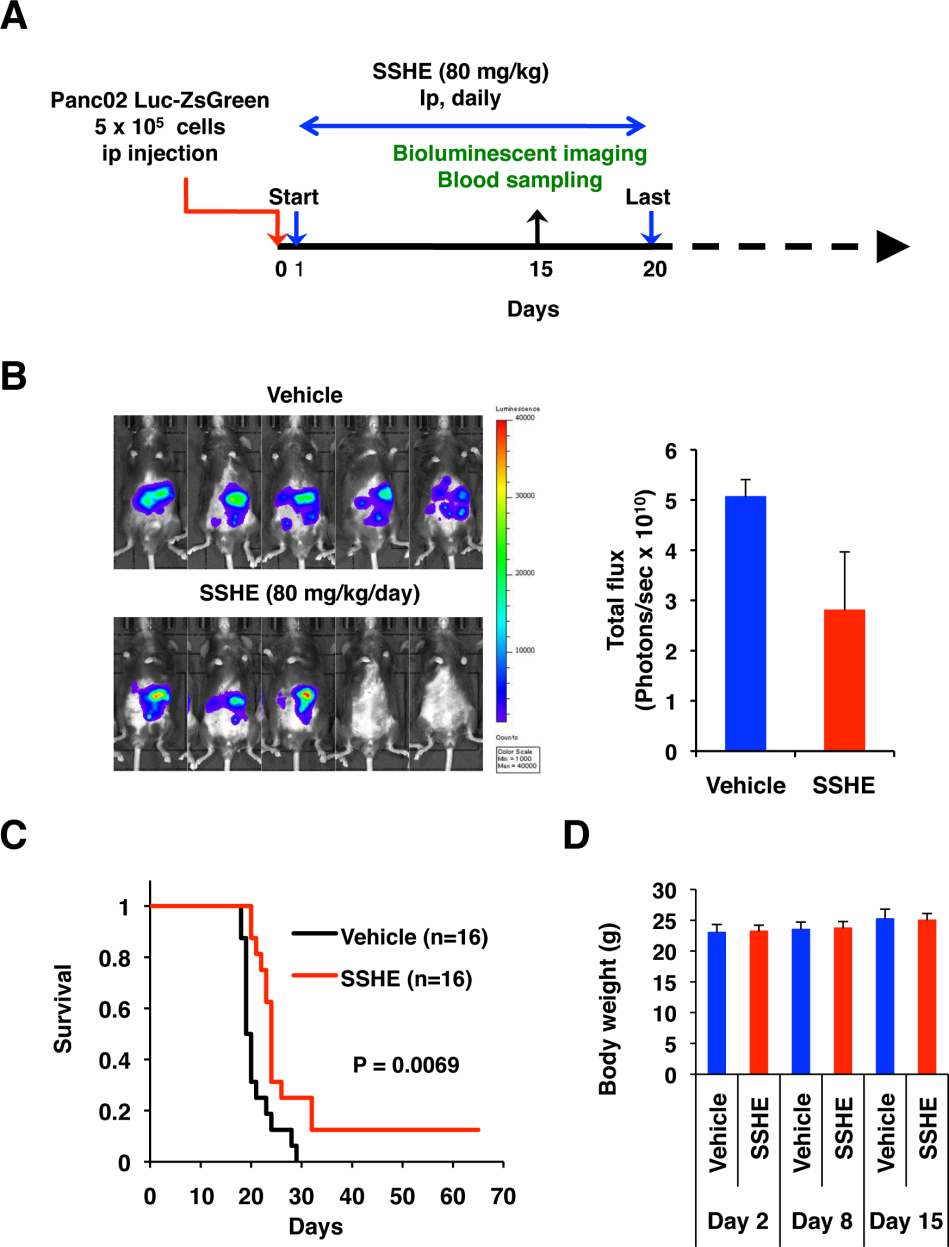
Antitumor effect of SSHE in the peritoneal dissemination model of Panc02 cells. (A) Experimental set-up. (B) Bioluminescence imaging on Day 15. Left, IVIS images. Right, Quantitation of photons. (C) Kaplan-Meier survival curve. (D) Body weight.

When Panc02-Luc-ZsGreen cells (5 x 10^5^ cells) were intraperitoneally transplanted into C57BL/6 mice, they tended to form disseminated nodules preferentially around the pancreas and on the peritoneal surface, and the mice developed ascites (approximately 2.4 ml/mouse when moribund) (S6 Fig). We examined the therapeutic efficacy of SSHE in this peritoneal dissemination model. We intraperitoneally administered SSHE at 80 mg/kg once daily for 20 days starting from Day 1 post-injection of Panc02 cells (Fig 6A). Bioluminescence imaging on Day 15 revealed that SSHE tended to suppress the growth of Panc02 cells (Fig 6B). Indeed, SSHE significantly prolonged the survival of mice (mean survival time; control group 20.9 days versus SSHE group 29.3 days, P = 0.0069 by log-rank test) (Fig 6C). Loss of body weight was not evident (Fig 6D).

To examine any toxicity of SSHE in more detail, hematological and biochemical blood tests were performed on Day 15. In a routine peripheral blood analysis, there were no differences in the levels of WBC, RBC, PLT, HGB, HCT, MCV, MCH, or MCHC between the control and the SSHE-treated groups (S7 Fig). The Glu levels were slightly higher in the SSHE-treated group than in the control group (P < 0.01). The T-Cho levels were not affected by SSHE. In terms of renal function, BUN levels in the SSHE-treated group were only slightly higher than those in the control group (P < 0.05). With respect to liver function, there were no significant differences in T-Bil, AST and ALT levels (S7 Fig). Overall, we did not observe serious abnormalities in the SSHE-treated mice.

**Fig 7:**
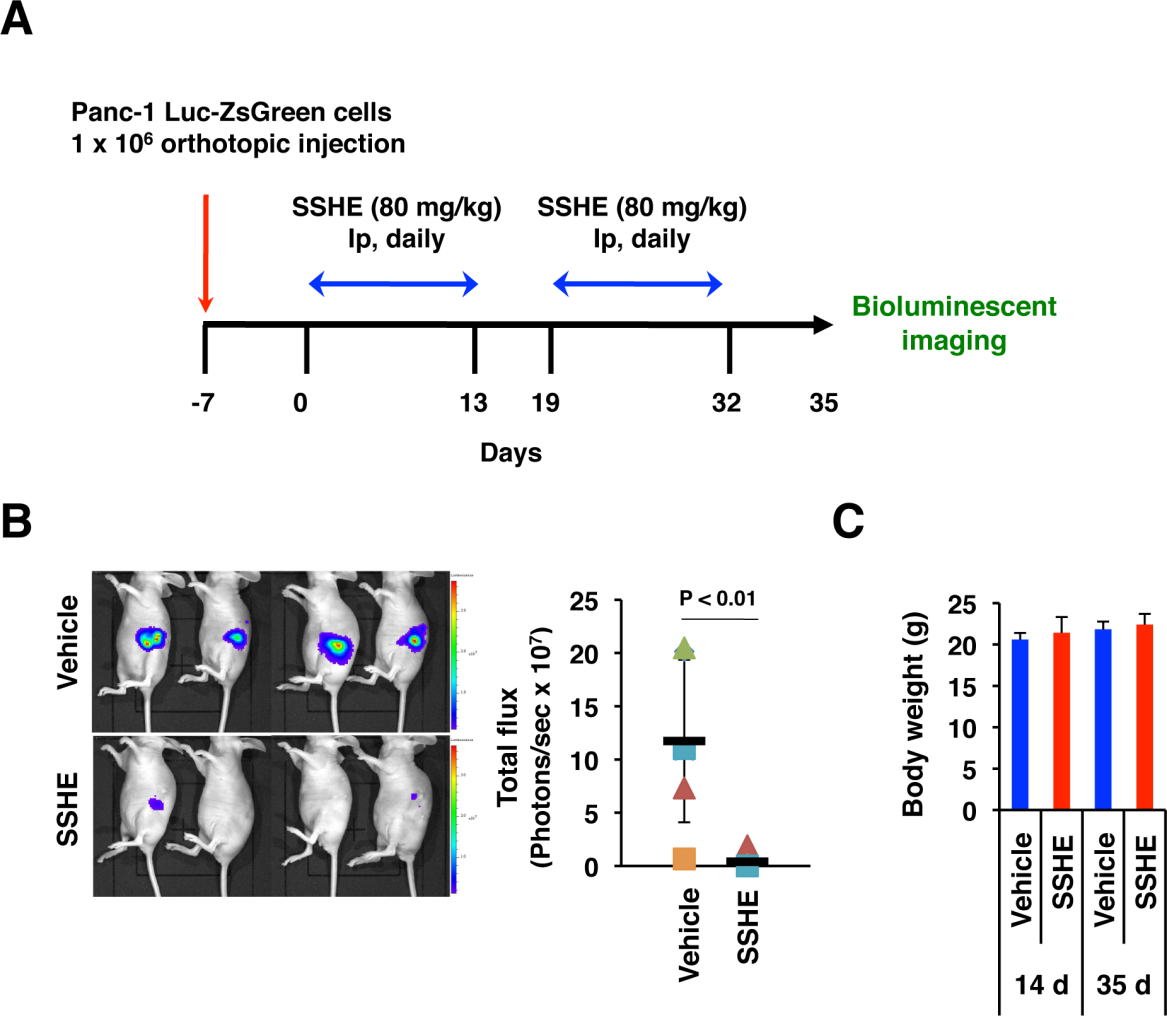
Antitumor effect of SSHE in the orthotopic model of Panc-1 cells. (A) Experimental set-up. (B) Bioluminescence imaging on Day 35. Left, IVIS images. Right, Quantitation of photons. (C) Body weight. Control group; n = 6, SSHE group; n = 5.

We also investigated the effect of intraperitoneal administration of SSHE on the tumor growth of orthotopically implanted Panc-1 cells. Panc-1-Luc-ZsGreen cells (1 x10^6^ cells) were injected into the pancreas of nude mice with Matrigel. One week after the injection, the mice were randomized (6 mice/group) and then treated with 80 mg/kg SSHE. Two 2-week periods of once-daily administration of SSHE were performed, with a 5-day resting phase in between treatment periods (Fig 7A). Unfortunately, a mouse in the treatment group showed splenomegaly of unknown cause during the first treatment period, so we excluded that mouse from the experiment. Bioluminescence imaging on Day 35 clearly showed that SSHE significantly suppressed the growth of Panc-1 cells (P < 0.01) without loss of body weight (Fig 7B and 7C).

Additionally, SSHE also suppressed tumor growth of subcutaneously transplanted mouse colon carcinoma LuM1 cells without loss of body weight (S8 Fig).

### [6]-Shogaol causes cell growth retardation and death of pancreatic cancer cells

To investigate which constituents of SSHE are active in retarding cell growth and inducing the death of Panc-1 cells, we examined the effect of [6]-shogaol and [6]-gingerol [14, 38–42]. The results showed that [6]-shogaol reduced the viability of Panc-1 cells with an IC50 value of ~18.8 μM (~5.2 μg/ml). It also caused cell growth retardation and cell death in other pancreatic cancer cell lines except CAPAN-2 cells and in other cancer cell lines (S9 Fig). [6]-Gingerol was effective only at very high doses, with an IC50 value of ~440 μM (~129.5 μg/ml) (S9 Fig). As was observed for SSHE, [6]-shogaol decreased the mitochondrial membrane potential, however, it did not activate caspase-3, and zVAD-fmk was ineffective in reversing [6]-shogaol-induced cell death (S10 Fig). Nuclear translocation of AIF was not observed, and necrostatin-1 did not show any effect (S10 Fig). On the other hand, [6]-shogaol increased the LC3-II/LC3-I ratio, and 3-methyladenine partly recovered the loss of viability caused by [6]-shogaol (S10 Fig). [6]-Shogaol increased ROS generation, and the loss of viability caused by [6]-shogaol was reversed by NAC (S10 Fig). Treatment of Panc-1 cells with [6]-shogaol resulted in the induction of nuclear shrinkage, cytoplasmic vacuolization, electron-dense mitochondria and empty cytoplasmic spaces (S11 Fig). In addition, ρ^0^P29 cells were resistant to [6]-shogaol-induced cell death compared with P29mtP29 cells (S5 Fig). Together, these results suggest that [6]-shogaol also induces the ROS-mediated autotic death of Panc-1 cells.

### Presence of potent cell death-inducing component(s) other than [6]-shogaol in SSHE

To investigate whether [6]-shogaol is the major cell death-inducing component in SSHE, we estimated the concentration of [6]-shogaol in SSHE. To this end, we carried out reversed-phase HPLC followed by the measurement of area corresponding to [6]-shogaol in SSHE. By extrapolating the area to the standard curve, the concentration of [6]-shogaol in SSHE was determined to be approximately 4.6 μg in 200 μg SSHE (S12 Fig). This concentration was not sufficient to achieve full activity of SSHE (Fig 1B), which suggested the presence of other active constituent(s). We then fractionated the constituents in SSHE and examined the cell death-inducing activity of each fraction. The results showed that potent activity was eluted in a few fractions, all of which were ahead of the [6]-gingerol fraction (S12 Fig), although the chemical composition of these fractions is presently unknown. [6]-Gingerol fractions showed moderate activity, most likely due to its high content in the extract. [6]-Shogaol fractions showed weak activity.

## Discussion

The present study demonstrated that the extract of Syussai ginger (SSHE) had potent growth-inhibitory and cell death-inducing activity against pancreatic cancer cells including Panc-1 cells. Normal cells such as HUVEC and HPAEpiC were relatively resistant to SSHE compared to Panc-1 cells. The extract was also effective under hypoxic conditions, which inevitably develop in all solid tumors to varying degrees and influence the resistance of tumor cells to radiotherapy and conventional chemotherapy [43, 44]. Among various cancers, pancreatic cancer is notorious for its unusually hypoxic microenvironments [45]. Our results showed that SSHE could induce the death of hypoxic Panc-1 cells, which may be a notable characteristic of SSHE for treating pancreatic cancers. In addition to pancreatic cancer, SSHE also induced a marked cell growth retardation and cell death in a variety of tumor cells, which supports the possibility of applying ginger extract and its active constituents for treating cancers.

After the treatment of Panc-1 cells with SSHE, the mitochondrial membrane potential was dramatically decreased and the number of annexin V-positive cells increased by 24 h after administration of SSHE. However, little increase in subG1 phase cells, fragmented nuclei and caspase-3 activation, all of which are features of apoptosis, were observed. Moreover, zVAD-fmk failed to rescue SSHE-induced cell death. Because annexin V stains both primary necroptotic and apoptotic cells [46] and mitochondria dysfunction can also be brought about by mitophagy, the autophagy-dependent elimination of mitochondria [47], we considered the possibility that SSHE-induced cell death was not due to apoptosis. Additionally, necroptosis was excluded because necrostatin-1 could not ameliorate the cell death induced by SSHE. We then explored the possibility of autophagic cell death and noticed many cytoplasmic vacuoles and LC3 puncta in the SSHE-treated Panc-1 cells at early stages. SSHE treatment dramatically increased the ratio of LC3-II/LC3-I and caused loss of SQSTM1/p62. SSHE activated AMPK and inhibited mTOR. 3-Methyladenine and chloroquine partially rescued SSHE-induced cell death. All of these data suggested that autophagy was occurring in the SSHE-treated cells. However, a close look at the SSHE-treated cells revealed several features that are not observed in classical type autophagy, including nuclear shrinkage, focal membrane rupture, electron-dense mitochondria, empty vacuoles and focal perinuclear swelling. Eventually, it appeared that these morphological features coincided well with the recently discovered form of cell death, “autosis” [36]. Thus, we presently consider that the SSHE-induced cell death of Panc-1 cells is mainly due to caspase-independent, autotic cell death rather than due to apoptosis and necroptosis.

Several lines of evidence indicate that ROS, more specifically mitochondria-derived ROS, are the inducer of autophagy upon various stresses such as nutrient deprivation [48–54]. Our data demonstrated that ROS production was suppressed in SSHE-treated Panc-1 cell at early stages. This may be owed to the antioxidant properties of ginger extract [5, 6]. However, prolonged treatment of the cells with SSHE caused a marked increase in ROS production. The ROS production was most likely the cause of SSHE-induced autotic cell death because NAC partially rescued the cell death induced by SSHE. This was further corroborated by the fact that ρ^0^P29 cells that produced little ROS were refractory to SSHE compared with P29mtP29 cells.

Our study in mice demonstrated that SSHE showed potent anticancer activity in both the peritoneal dissemination model and the orthotopic model. It also suppressed the tumor growth of subcutaneously implanted mouse colon carcinoma cells. Notably, apparent severe adverse effects, such as loss of body weight and low blood cell counts, were not evident in the SSHE-treated mice, although relatively minor abnormalities in the blood Glu and BUN levels were observed. Other side effects, such as diarrhea, constipation, or hair loss, also did not occur. Therefore, even at such a high tested dose, SSHE seemed to be safe and tolerable. Although it is difficult to extrapolate our observations to humans, a recent pilot clinical study demonstrated that oral intake of 2.0 g/day whole ginger extract (the materials extracted with 50% ethanol) for 28 days was tolerable and safe in humans [18]. Additionally, it has recently been reported that oral administration of whole ginger extract (100 mg/kg) was effective in suppressing xenografted PC-3 prostate cancer growth without any toxicity [15]. Thus, treatment with whole ginger extract may be beneficial for cancer patients.

[6]-Shogaol and [6]-gingerol are the major components of ginger extract, and both have been shown to exhibit anti-proliferative and apoptosis-inducing activities in tumor cells [55, 56]. Our data showed that, although [6]-gingerol had cell growth-inhibitory activity at very high concentrations, [6]-shogaol exerted potent activity at relatively low concentrations. However, the content of [6]-shogaol in SSHE was not sufficient enough to exhibit cell death-inducing activity. To search for other active component(s), reversed-phase HPLC followed by fractionation revealed three major peaks showing cell death-inducing activity; in the order of elution, the first peak had the most potent activity, and the second and the third peaks corresponded to the elution position of [6]-gingerol and [6]-shogaol, respectively. Identification of the component(s) in the first peak is currently under way.

In conclusion, the present study demonstrated that ginger extract inhibited cell proliferation and subsequently induced the autotic death of pancreatic cancer Panc-1 cells. The extract suppressed tumor growth without serious adverse effects in a Panc02 peritoneal dissemination mouse model and Panc-1 xenografted mice when administered intraperitoneally. Our results suggest that whole ginger extract or its constituents may have clinical implications for therapeutic intervention against pancreatic cancer.

## Supporting Information

S1 Fig Effect of SSHE on the viability of various tumor cells. The cells were treated with various concentrations of SSHE for 42 h. Cell viability was assessed by the MTT assay. Bars, SD.
(TIF)

S2 Fig Involvement of AIF and necroptosis in SSHE-induced cell death. (A) Apoptosis-inducing factor (AIF) staining. Panc-1 cells treated with 200 μg/ml SSHE for 28 h were fixed, and immunostained with anti-AIF antibody. The cells were counterstained with DAPI. Bar, 100 μm. (B) Effect of necrostatin-1 on SSHE-induced cell death. Panc-1 cells were treated with 200 μg/ml SSHE in the presence or absence of 100 μM necrostain-1 (Nec) for 38 h. Cell viability was assessed by the MTT assay. Bars, SD.
(TIF)

S3 Fig ROS production in SSHE-treated Panc-1 cells. Panc-1 cells treated with vehicle alone, 100 μg/ml SSHE for 10 h were stained with 10 μM H2DCFDA for 10 min and immediately observed under a confocal laser microscope or subjected to cytometry.
(TIF)

S4 Fig Mitochondrial superoxide production in SSHE-treated Panc-1 cells. Panc-1 cells treated with vehicle alone, 100 μg/ml or 200 μg/ml SSHE for 20 h were stained with 5 μM MitoSOX Red for 10 min and immediately observed under a confocal laser microscope or subjected to cytometry.
(TIF)

S5 Fig Effect of SSHE on the viability of mtDNA-less mouse lung carcinoma cells. (A) ROS production. ρ^0^P29 cells and P29mtP29 cells were treated with 200 μg/ml SSHE for 20 h, stained with 10 μM H2DCFDA for 10 min and immediately subjected to cytometry. (B) Effect of SSHE on cell viability. The cells were treated with various concentrations of SSHE or [6]-shogaol for 42 h. Cell viability was assessed by the MTT assay. Bars, SD.
(TIF)

S6 Fig Peritoneal dissemination model of Panc02 cells. C57BL/6 mice were intraperitoneally inoculated with 5 x 10^5^ Panc02-Luc-ZsGreen cells. On Day 10, bioluminescence images were obtained. On day 24, the mice were euthanized and autopsied. Ascites fluid was also collected. Arrows indicate disseminated nodules.
(TIF)

S7 Fig Hematological and biochemical blood test of SSHE-treated mice. (A) Blood test. n = 6. (B) Biological test. n = 6. NS, not significant.
(TIF)

S8 Fig Effect of administration of SSHE on tumor growth of colon carcinoma cells. Mouse colon carcinoma LuM1 cells (3 x 10^5^ cells) were subcutaneously implanted in Balb/c mice (n = 6). SSHE (80 mg/kg) was intraperitoneally administered once daily. (A) Tumor growth. Bars, SD. (B) Tumor weight. (C) Body weight.
(TIF)

S9 Fig Effect of [6]-shogaol and [6]-gingerol on cell death of pancreatic cancer cells. (A) Cell viability. Panc-1 cells were treated with vehicle alone or various concentrations of [6]-shogaol or [6]-gingerol for 42 h. (B) Cell viability of various pancreatic cancer cell lines treated with [6]-shogaol. The cell lines were treated with vehicle alone or various concentrations of [6]-shogaol for 42 h. (C) Effect of [6]-shogaol on the viability of various tumor cells. The cells were treated with various concentrations of [6]-shogaol for 42 h. Cell viability was assessed by the MTT assay. Bars, SD.
(TIF)

S10 Fig Effect of [6]-shogaol on Panc-1 cells. (A) Mitochondrial membrane potential. Panc-1 cells were treated with vehicle alone, 25 μM SSHE or 50 μM SSHE for 22 h and then subjected to the JC-1 assay. (B) Caspase-3 activation. Panc-1 cells were incubated with 50 μM [6]-shogaol for various periods. The cell lysates were subjected to Western blot analysis with anti-caspase 3 antibody. (C) Effect of zVAD-fmk on [6]-shogaol-induced cell death. Panc-1 cells were incubated with 50 μM [6]-shogaol in the presence or absence of 10 μM zVAD-fmk (zVAD) for 42 h. Cell viability was assessed by the MTT assay. Bars; SD. (D) Apoptosis-inducing factor (AIF) staining. Panc-1 cells treated with 25 μM [6]-shogaol for 28 h were fixed and immunostained with anti-AIF antibody. The cells were counterstained with DAPI. Bar, 100 μm. (E) Effect of necrostatin-1 on [6]-shogaol-induced cell death. Panc-1 cells were treated with 25 μM [6]-shogaol in the presence or absence of 100 μM necrostain-1 (Nec) for 42 h. Cell viability was assessed by the MTT assay. Bars, SD. (F) Effect of [6]-shogaol on the conversion of LC3-I to LC3-II. Panc-1 cells were treated with 50 μM [6]-shogaol for up to 24 h. The cell lysates were subjected to Western blot analysis with anti-LC3 antibody. β-Actin served as a loading control. (G) Effect of 3-methyladenine (3-MA) on [6]-shogaol-induced cell death. Panc-1 cells were incubated with 50 μM [6]-shogaol in the presence of 10 μM 3-MA (left) for 42 h. Cell viability was assessed by the MTT assay. Bars; SD. (H) ROS production in [6]-shogaol-treated Panc-1 cells. Panc-1 cells treated with vehicle alone, 25 μM or 50 μM [6]-shogaol for 20 h were stained with 10 μM H2DCFDA for 10 min and immediately subjected to cytometry. (I) Effect of N-acetylcysteine (NAC) on SSHE-induced cell death. Panc-1 cells were treated with 200 μg/ml SSHE in the presence or absence of 10 mM NAC for 42 h. Cell viability was assessed by a trypan blue dye exclusion test. Bars; SD.
(TIF)

S11 Fig Morphological features of [6]-shogaol-treated Panc-1 cells. (A) Phase-contrast images and DAPI staining. Panc-1 cells were treated with 50 μM [6]-shogaol for 40 h. Bars; 100 μm. (B) Phase contrast. Panc-1 cells were treated with 50 μg/ml SSHE for 42 h. Bars; 50 μm. (C) Ultrastructure. Panc-1 cells treated with 50 μM [6]-shogaol for 28 h were processed for TEM analysis. Original magnification, x4,500.
(TIF)

S12 Fig Quantitation of the concentration of [6]-shogaol in SSHE. (A) The standard curve was made by measuring the area of the reversed-phase HPLC profile after loading a known quantity of [6]-shogaol. SSHE (200 μg) was separated by HPLC, and the area corresponding to the position of 6-shogaol was measured. The amount of [6]-shogaol in SSHE was determined by extrapolating the area to the standard curve. (B) Fractionation of SSHE by reversed-phase HPLC. After fractionation, each fraction was evaporated and dissolved in DMSO, which was then added to Panc-1 cells. Cell viability was determined by the MTT assay after a 38-h incubation. The red arrowhead indicates the fractions showing potent cell death-inducing activity.
(TIF)

S13 Fig Full size images of the Western blots presented in Fig 3. (TIF)
