# Supplementary figures and images for: Anticancer Effect of Ginger Extract against Pancreatic Cancer Cells Mainly through Reactive Oxygen Species-Mediated Autotic Cell Death
Source: PLoS One. 2015 May 11;10(5):e0126605. doi: 10.1371/journal.pone.0126605 (PMC4427290; doi:10.1371/journal.pone.0126605)

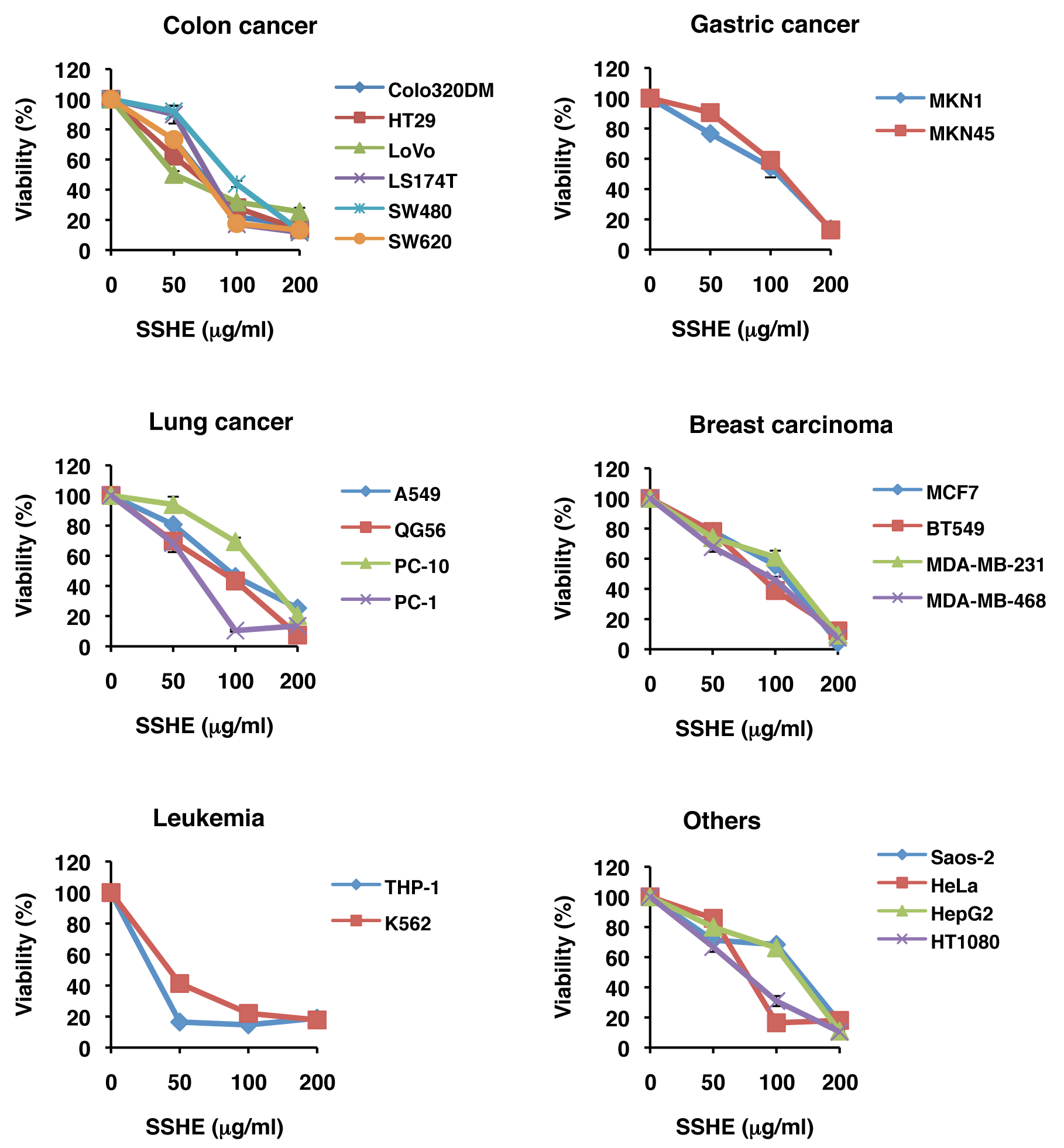

Supplement: S1 Fig — The cells were treated with various concentrations of SSHE for 42 h. Cell viability was assessed by the MTT assay. Bars, SD. (TIF) [file pone.0126605.s001.tif]

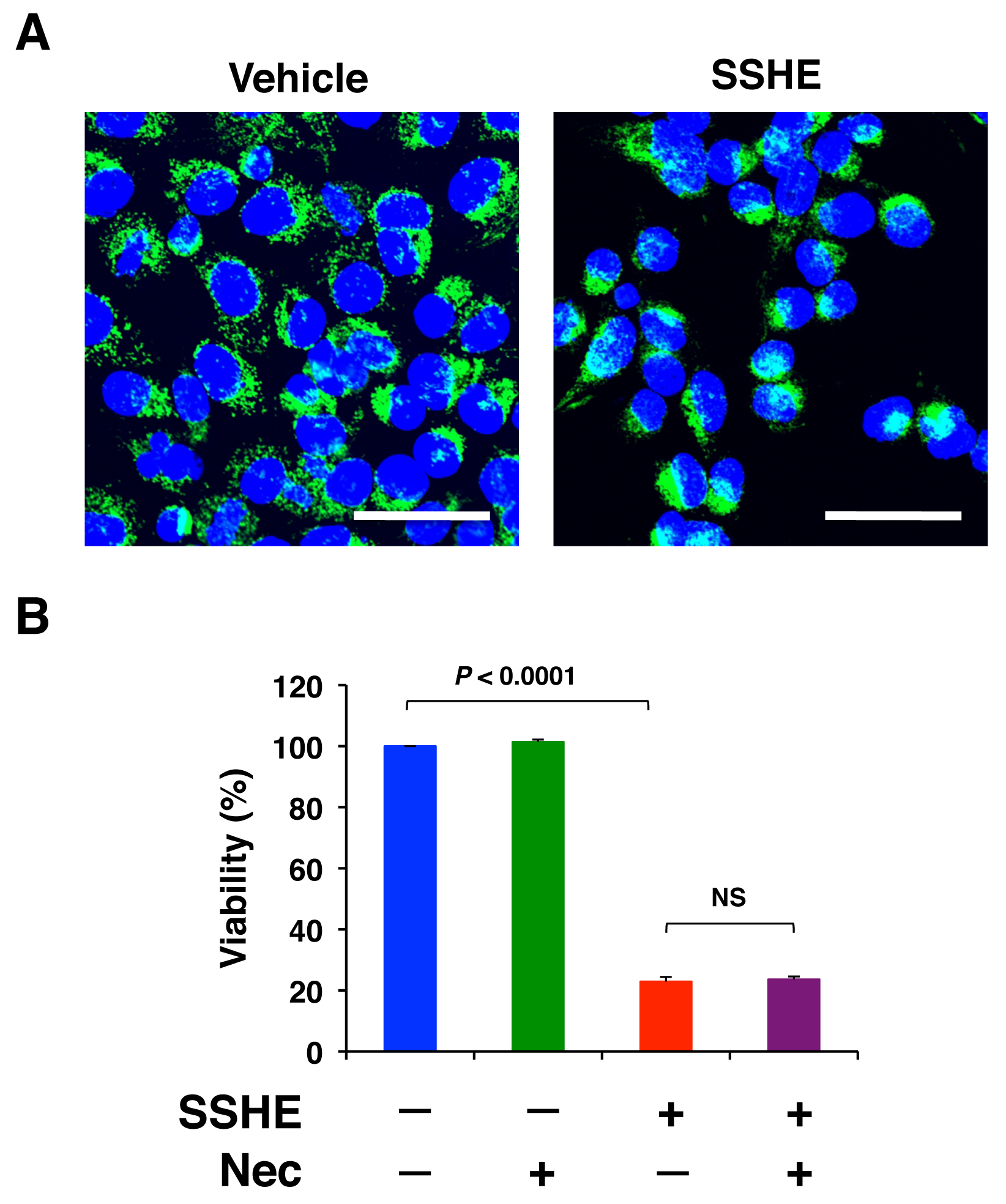

Supplement: S2 Fig — (A) Apoptosis-inducing factor (AIF) staining. Panc-1 cells treated with 200 μg/ml SSHE for 28 h were fixed, and immunostained with anti-AIF antibody. The cells were counterstained with DAPI. Bar, 100 μm. (B) Effect of necrostatin-1 on SSHE-induced cell death. Panc-1 cells were treated with 200 μg/ml SSHE in the presence or absence of 100 μM necrostain-1 (Nec) for 38 h. Cell viability was assessed by the MTT assay. Bars, SD. (TIF) [file pone.0126605.s002.tif]

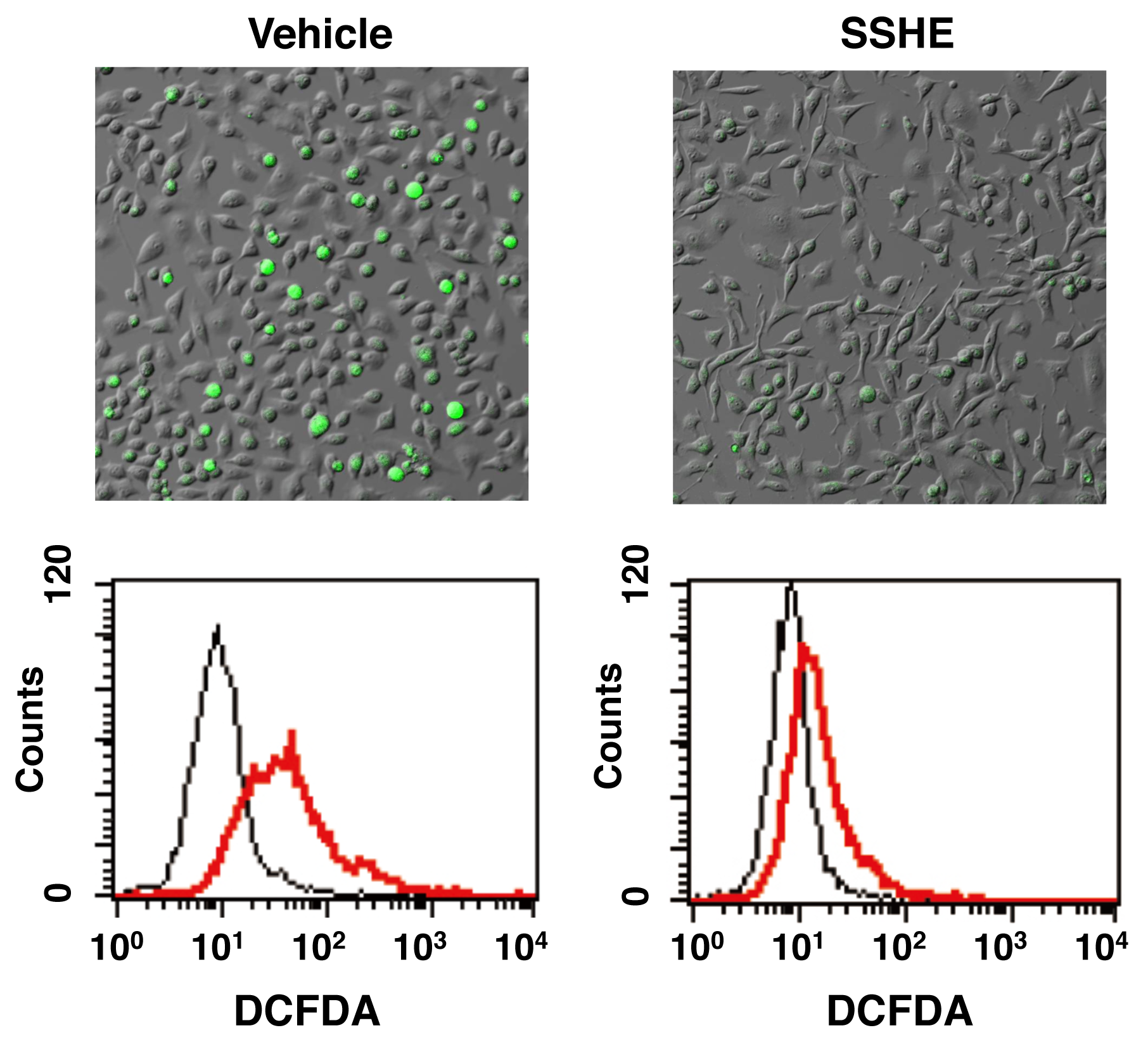

Supplement: S3 Fig — Panc-1 cells treated with vehicle alone, 100 μg/ml SSHE for 10 h were stained with 10 μM H2DCFDA for 10 min and immediately observed under a confocal laser microscope or subjected to cytometry. (TIF) [file pone.0126605.s003.tif]

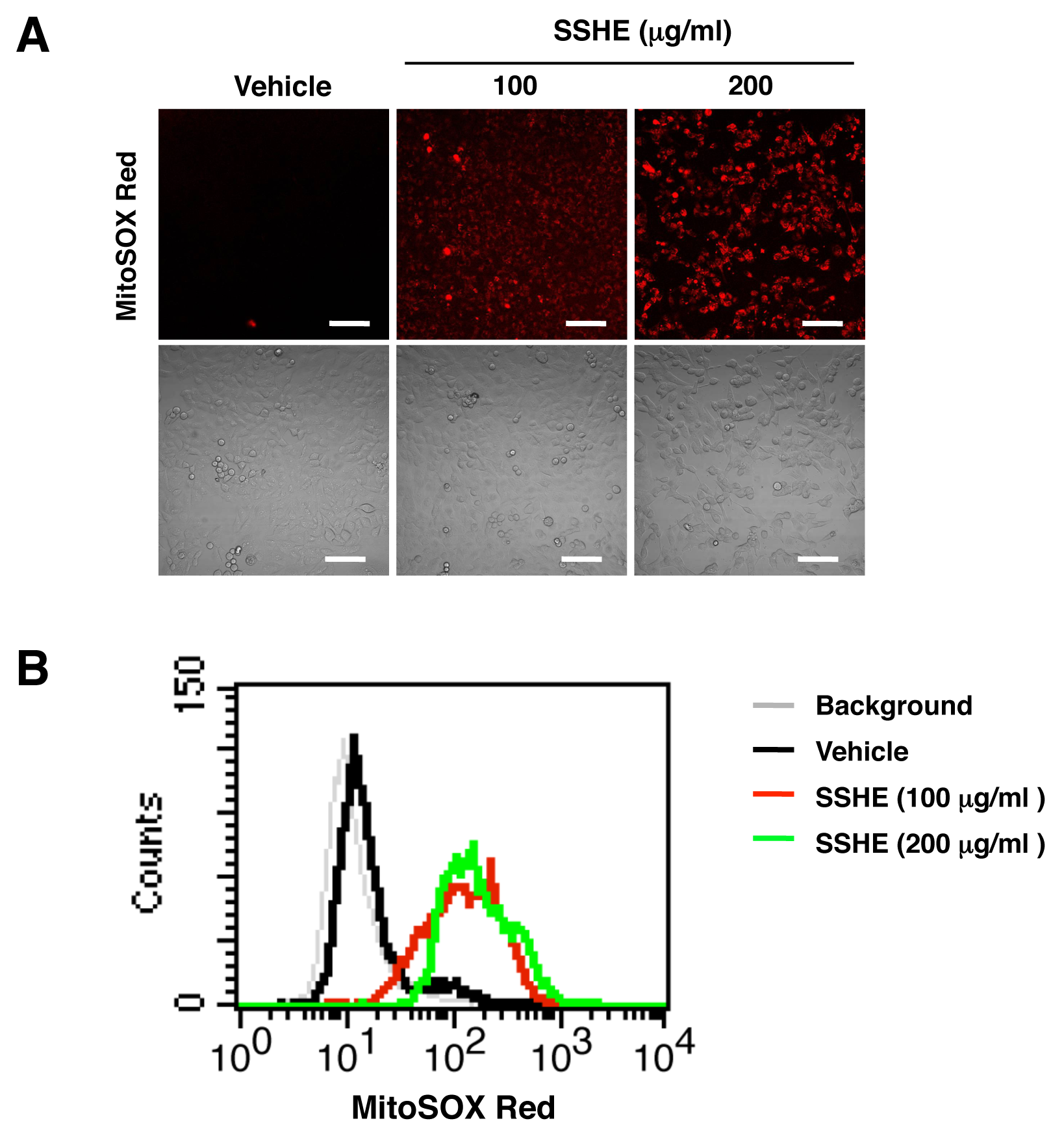

Supplement: S4 Fig — Panc-1 cells treated with vehicle alone, 100 μg/ml or 200 μg/ml SSHE for 20 h were stained with 5 μM MitoSOX Red for 10 min and immediately observed under a confocal laser microscope or subjected to cytometry. (TIF) [file pone.0126605.s004.tif]

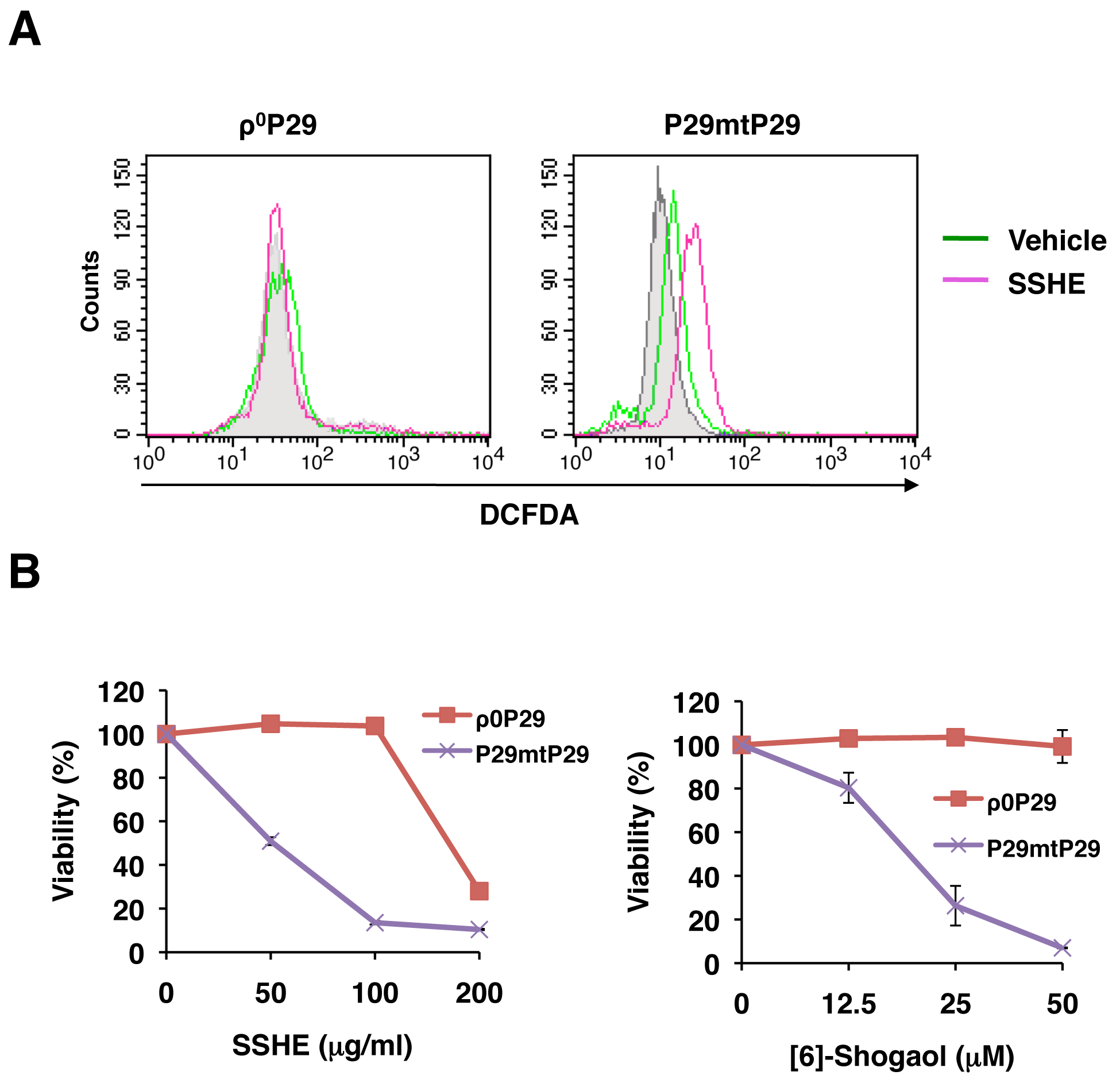

Supplement: S5 Fig — (A) ROS production. ρ0P29 cells and P29mtP29 cells were treated with 200 μg/ml SSHE for 20 h, stained with 10 μM H2DCFDA for 10 min and immediately subjected to cytometry. (B) Effect of SSHE on cell viability. The cells were treated with various concentrations of SSHE or [6]-shogaol for 42 h. Cell viability was assessed by the MTT assay. Bars, SD. (TIF) [file pone.0126605.s005.tif]

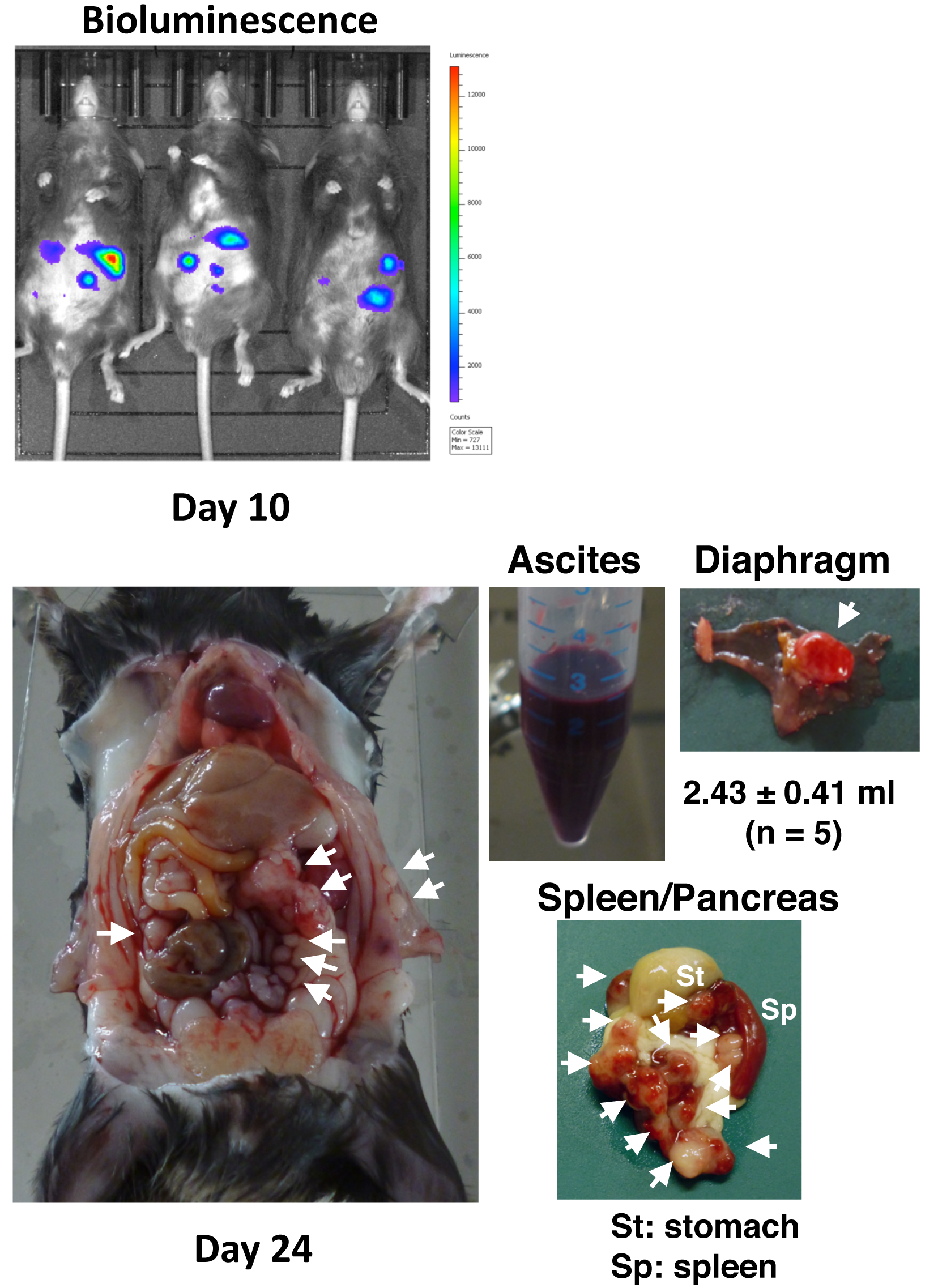

Supplement: S6 Fig — C57BL/6 mice were intraperitoneally inoculated with 5 x 10^5^ Panc02-Luc-ZsGreen cells. On Day 10, bioluminescence images were obtained. On day 24, the mice were euthanized and autopsied. Ascites fluid was also collected. Arrows indicate disseminated nodules. (TIF) [file pone.0126605.s006.tif]

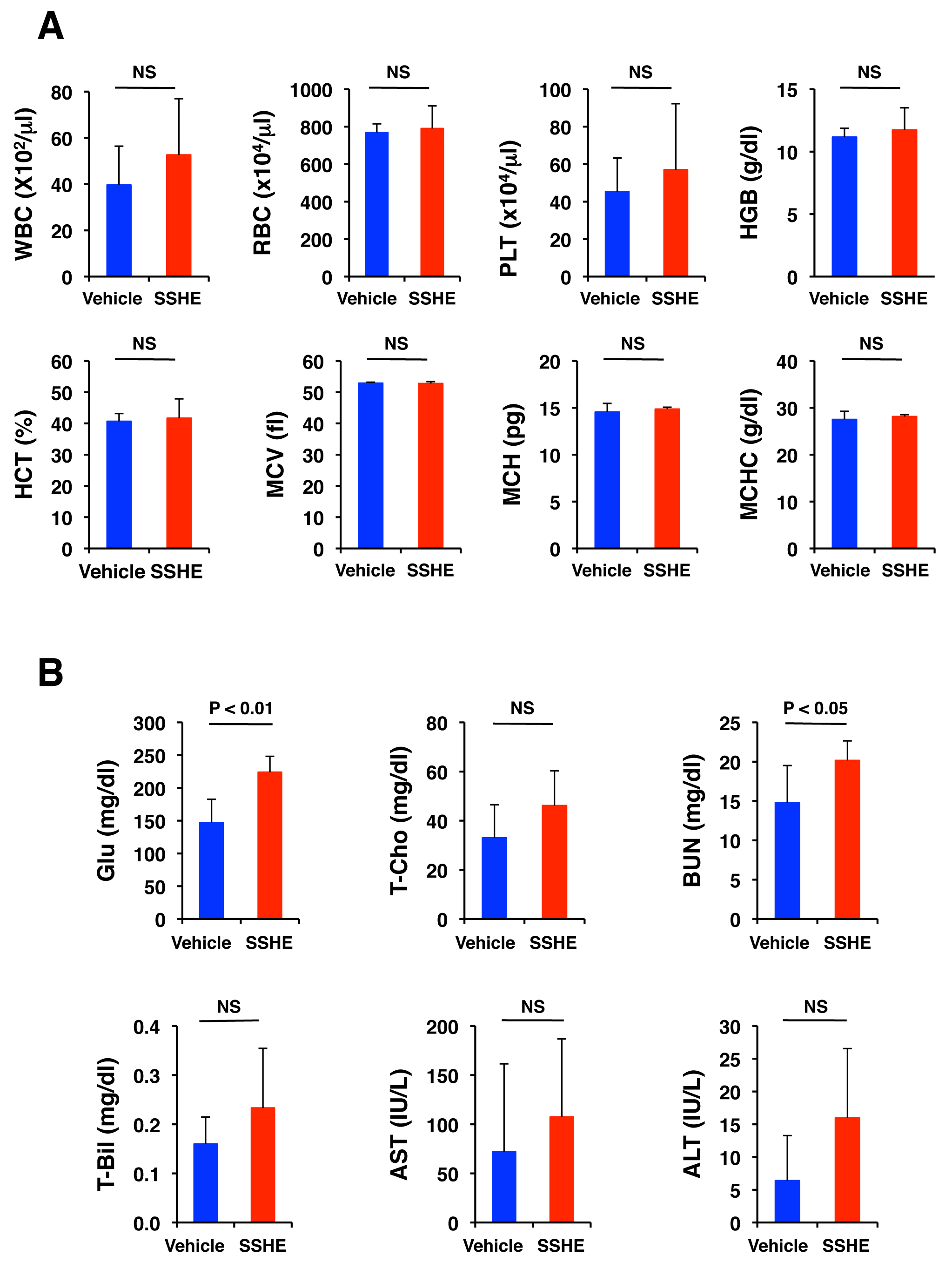

Supplement: S7 Fig — (A) Blood test. n = 6. (B) Biological test. n = 6. NS, not significant. (TIF) [file pone.0126605.s007.tif]

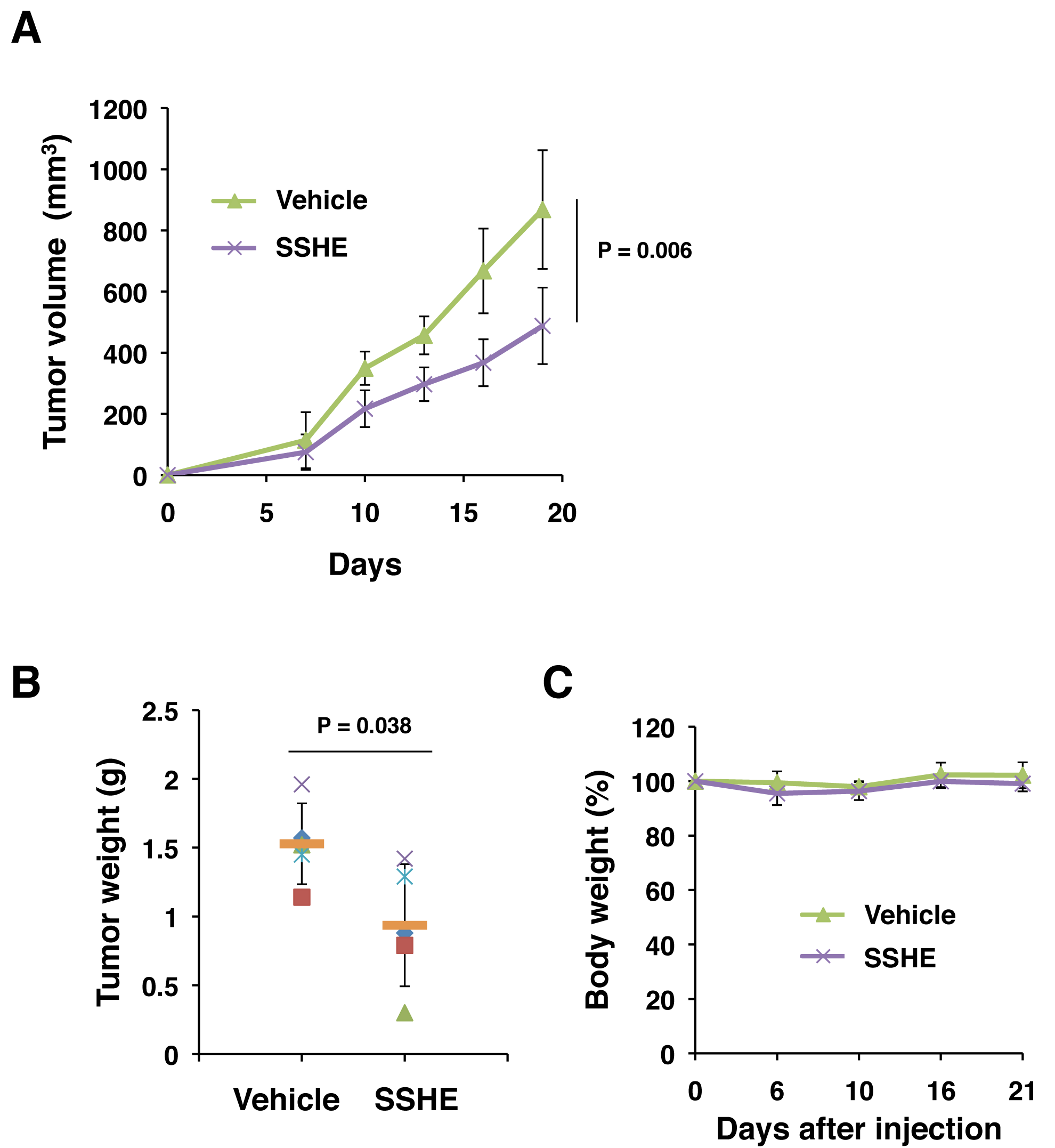

Supplement: S8 Fig — Mouse colon carcinoma LuM1 cells (3 x 10^5^ cells) were subcutaneously implanted in Balb/c mice (n = 6). SSHE (80 mg/kg) was intraperitoneally administered once daily. (A) Tumor growth. Bars, SD. (B) Tumor weight. (C) Body weight. (TIF) [file pone.0126605.s008.tif]

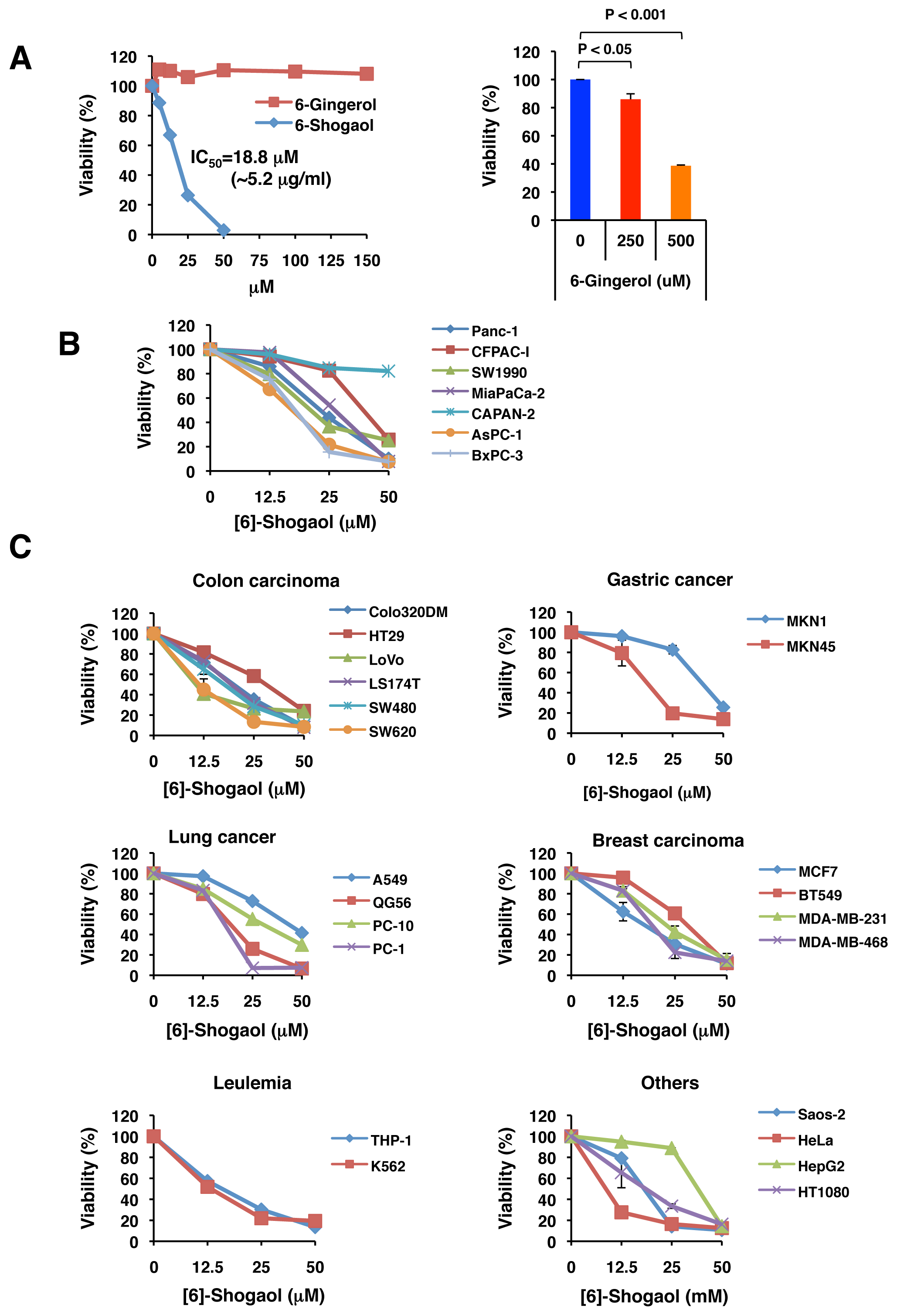

Supplement: S9 Fig — (A) Cell viability. Panc-1 cells were treated with vehicle alone or various concentrations of [6]-shogaol or [6]-gingerol for 42 h. (B) Cell viability of various pancreatic cancer cell lines treated with [6]-shogaol. The cell lines were treated with vehicle alone or various concentrations of [6]-shogaol for 42 h. (C) Effect of [6]-shogaol on the viability of various tumor cells. The cells were treated with various concentrations of [6]-shogaol for 42 h. Cell viability was assessed by the MTT assay. Bars, SD. (TIF) [file pone.0126605.s009.tif]

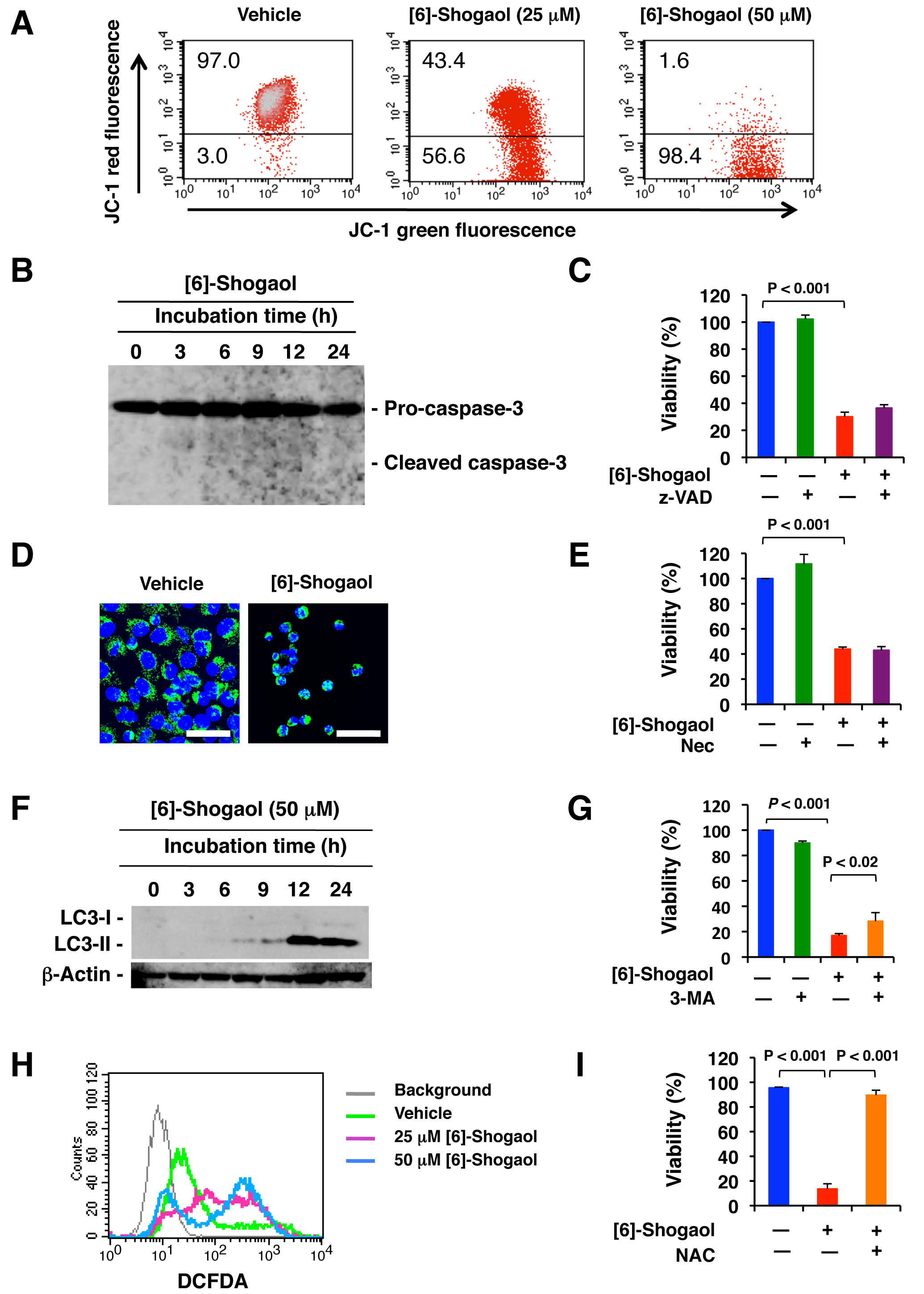

Supplement: S10 Fig — (A) Mitochondrial membrane potential. Panc-1 cells were treated with vehicle alone, 25 μM SSHE or 50 μM SSHE for 22 h and then subjected to the JC-1 assay. (B) Caspase-3 activation. Panc-1 cells were incubated with 50 μM [6]-shogaol for various periods. The cell lysates were subjected to Western blot analysis with anti-caspase 3 antibody. (C) Effect of zVAD-fmk on [6]-shogaol-induced cell death. Panc-1 cells were incubated with 50 μM [6]-shogaol in the presence or absence of 10 μM zVAD-fmk (zVAD) for 42 h. Cell viability was assessed by the MTT assay. Bars; SD. (D) Apoptosis-inducing factor (AIF) staining. Panc-1 cells treated with 25 μM [6]-shogaol for 28 h were fixed and immunostained with anti-AIF antibody. The cells were counterstained with DAPI. Bar, 100 μm. (E) Effect of necrostatin-1 on [6]-shogaol-induced cell death. Panc-1 cells were treated with 25 μM [6]-shogaol in the presence or absence of 100 μM necrostain-1 (Nec) for 42 h. Cell viability was assessed by the MTT assay. Bars, SD. (F) Effect of [6]-shogaol on the conversion of LC3-I to LC3-II. Panc-1 cells were treated with 50 μM [6]-shogaol for up to 24 h. The cell lysates were subjected to Western blot analysis with anti-LC3 antibody. β-Actin served as a loading control. (G) Effect of 3-methyladenine (3-MA) on [6]-shogaol-induced cell death. Panc-1 cells were incubated with 50 μM [6]-shogaol in the presence of 10 μM 3-MA (left) for 42 h. Cell viability was assessed by the MTT assay. Bars; SD. (H) ROS production in [6]-shogaol-treated Panc-1 cells. Panc-1 cells treated with vehicle alone, 25 μM or 50 μM [6]-shogaol for 20 h were stained with 10 μM H2DCFDA for 10 min and immediately subjected to cytometry. (I) Effect of N-acetylcysteine (NAC) on SSHE-induced cell death. Panc-1 cells were treated with 200 μg/ml SSHE in the presence or absence of 10 mM NAC for 42 h. Cell viability was assessed by a trypan blue dye exclusion test. Bars; SD. (TIF) [file pone.0126605.s010.tif]

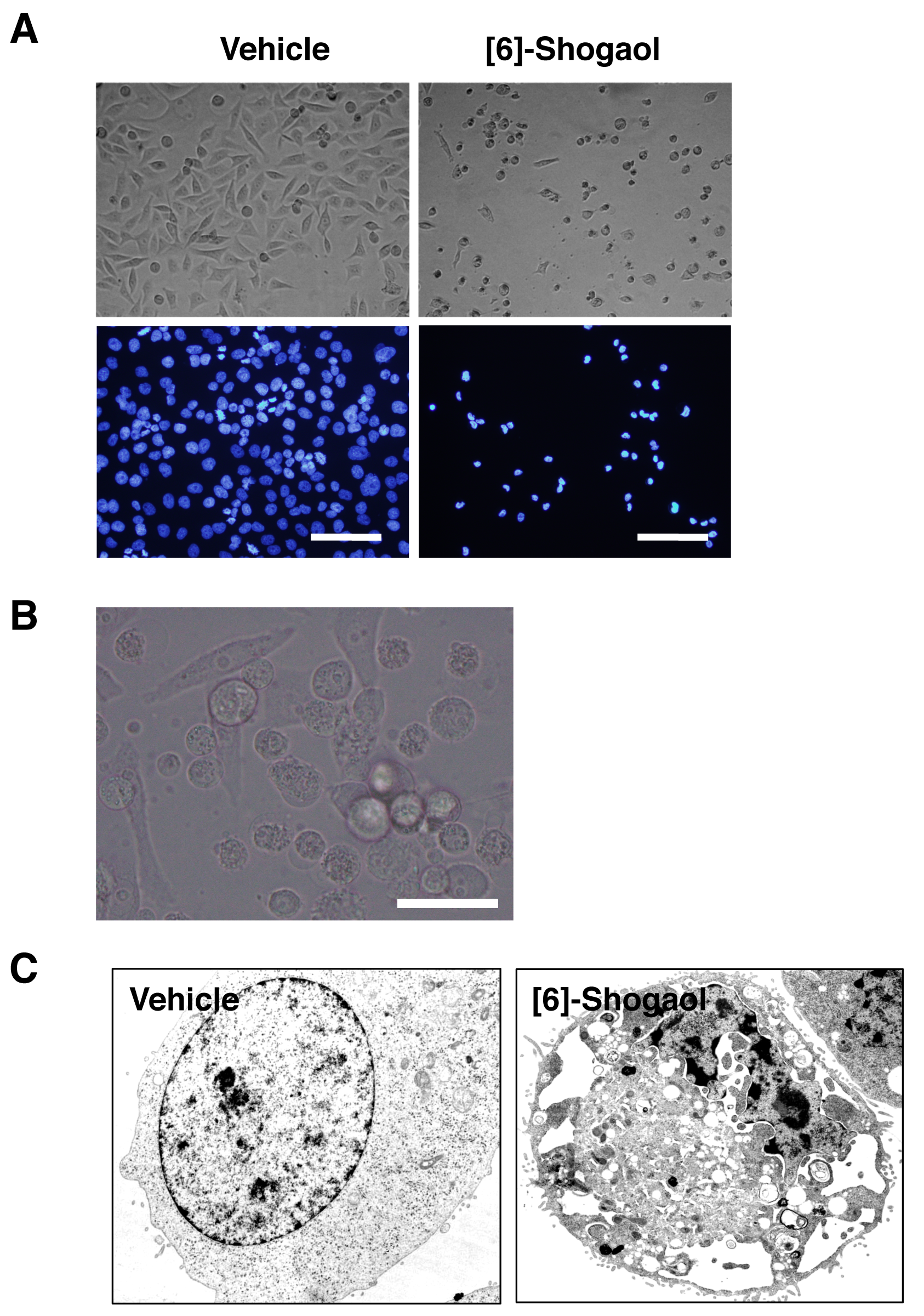

Supplement: S11 Fig — (A) Phase-contrast images and DAPI staining. Panc-1 cells were treated with 50 μM [6]-shogaol for 40 h. Bars; 100 μm. (B) Phase contrast. Panc-1 cells were treated with 50 μg/ml SSHE for 42 h. Bars; 50 μm. (C) Ultrastructure. Panc-1 cells treated with 50 μM [6]-shogaol for 28 h were processed for TEM analysis. Original magnification, x4,500. (TIF) [file pone.0126605.s011.tif]

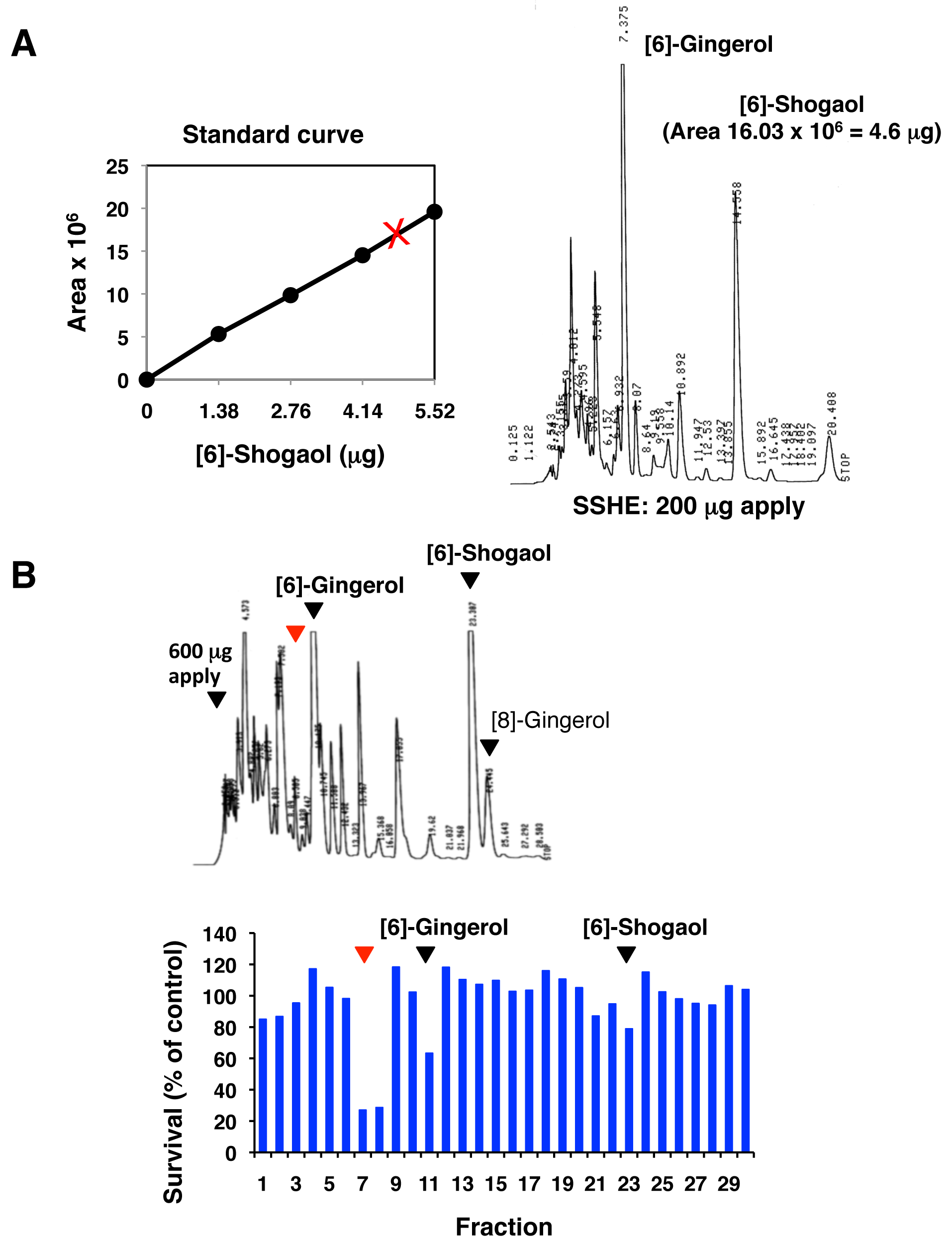

Supplement: S12 Fig — (A) The standard curve was made by measuring the area of the reversed-phase HPLC profile after loading a known quantity of [6]-shogaol. SSHE (200 μg) was separated by HPLC, and the area corresponding to the position of 6-shogaol was measured. The amount of [6]-shogaol in SSHE was determined by extrapolating the area to the standard curve. (B) Fractionation of SSHE by reversed-phase HPLC. After fractionation, each fraction was evaporated and dissolved in DMSO, which was then added to Panc-1 cells. Cell viability was determined by the MTT assay after a 38-h incubation. The red arrowhead indicates the fractions showing potent cell death-inducing activity. (TIF) [file pone.0126605.s012.tif]

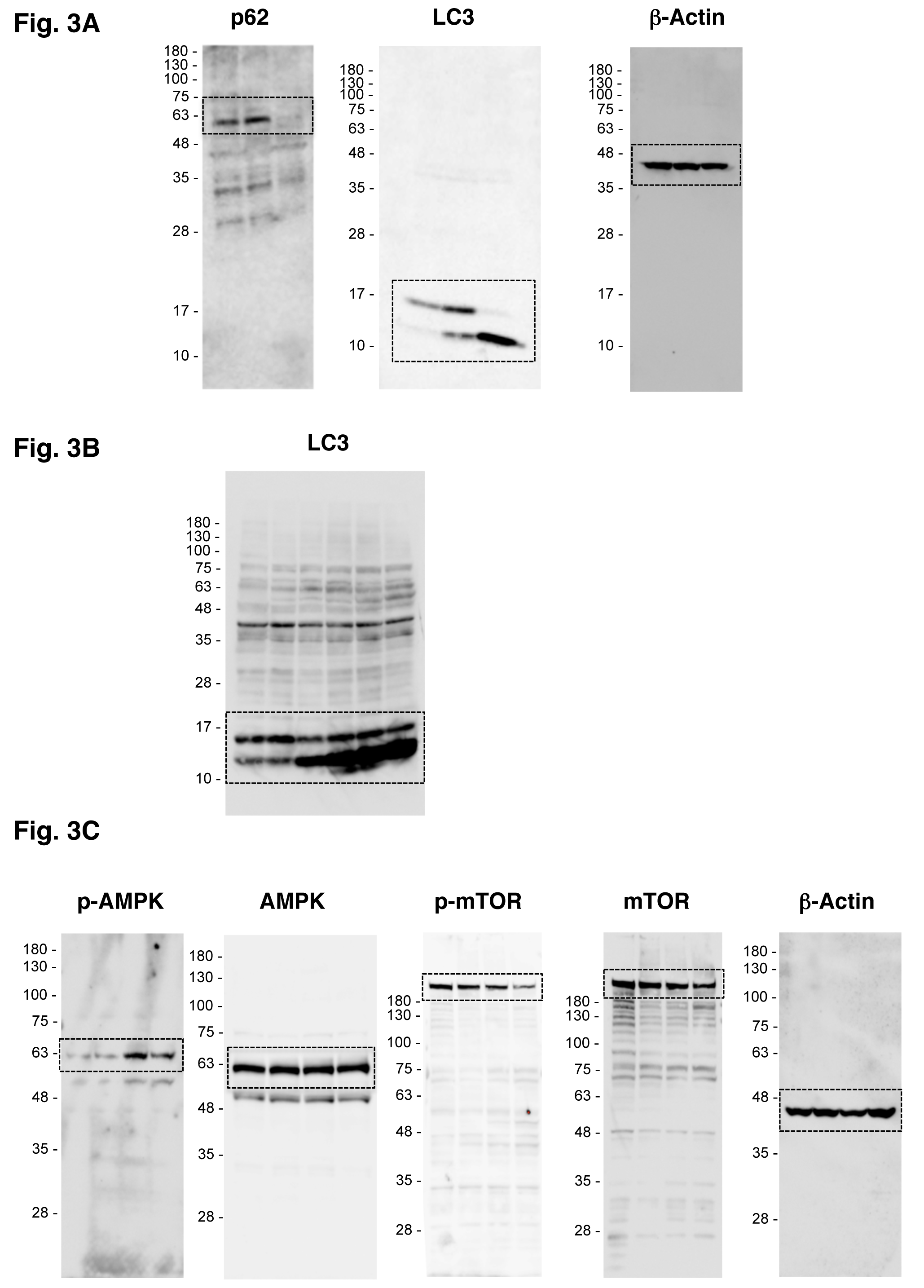

Supplement: S13 Fig — (TIF) [file pone.0126605.s013.tif]
